# Assessing distribution changes of selected native and alien invasive plant species under changing climatic conditions in Nyeri County, Kenya

**DOI:** 10.1371/journal.pone.0275360

**Published:** 2022-10-03

**Authors:** Julius Maina Waititu, Charles Ndegwa Mundia, Arthur W. Sichangi

**Affiliations:** Institute of Geomatics, GIS and Remote Sensing, Dedan Kimathi University of Technology, Nyeri, Kenya; USDA Forest Service Southern Research Station, UNITED STATES

## Abstract

The role of climate change in enhancing bio-invasions in natural environments needs to be assessed to provide baseline information for effective species management and policy formulations. In this study, potential habitat suitability maps were generated through Ecological Niche Modeling for five problematic alien and native species in current and future climate simulations for the periods 2050s and 2070s under RCP2.6, RCP4.5, and RCP8.5 emission scenarios. Projected current binary suitability maps showed that 67%, 40%, 28%, 68%, and 54% of the total study area ~ 3318 Km^2^ is suitable for *C*. *decapetala*, *L*. *camara*, *O*. *stricta*, *S*. *didymobotrya* and *S*. *campylacanthum* species, respectively. Assuming unlimited species dispersal, two of these species, *C*. *decapetala* and *S*. *didymobotrya*, were observed to have consistent gradual increase in potential habitats and no habitat losses under the three RCPs by the end of the 2050 and 2070 future periods. The highest recorded relative potential habitat increase was observed for *O*. *stricta* at ~205% under RCP2.6 and ~223% under RCP8.5. Although *L*. *camara* and *O*. *stricta* were observed to have habitat losses, the losses will be very low as compared to that of *S*. *campylacanthum*. *L*. *camara* and *O*. *stricta* relative habitat losses were predicted to be between ~1% under RCP2.6 to ~4.5% under RCP8.5 by 2070 while that of *S*. *campylacanthum* was between ~50% under RCP2.6 to ~68% under RCP8.5 by the year 2070. From this study we conclude that the target study species are expected to remain a big threat to inhabited areas as well as biodiversity hotspot areas especially in the Mt. Kenya and the Aberdare forest and national park reserves under climate change. The information generated through this study can be used to inform policy on prioritizing management of these species and subsequent determination of their absolute distributions within the area.

## Introduction

According to Richardson *et al*. [1], naturalized plants are alien plants that have consistently reproduced with no human intervention over many growth periods while invasive plants are naturalized plants that are able to produce many reproductive offspring at considerable distances from parent plants. Many alien invasive plant species have profound negative impacts on forest resources, water resources, and agricultural ecosystems [2–4]. In some instances, alien invasive species may be beneficial to new environments e.g. supporting local fauna in their habitats, reducing carbon footprint, and provision of firewood [5].

Invasive species are considered a nuisance if they are expanding in range and causing habitat transformations [6]. Although proliferation of alien invasive species in new environments may be attributed to lack of natural enemies inhibiting their survival [7], changes in climatic conditions such as temperature and precipitation may render any of the alien taxa to an extinction trajectory or may enable its spread and survival [1]. In most cases, alien invasive species’ wide tolerance to changing environmental conditions from historical norms gives them a competitive edge over less tolerant native species in persistence and expansion in geographical range [8, 9]. A study by Early *et al*. [10] reported that 15% of land area in parts of African, South American and Asian countries with low-Human Development Index values have a high risk of alien species invasions. In these regions, the establishment of these species is mostly enhanced by overlapping factors such as total imports and airport capacity, increased agricultural practices, and climate change-driven distribution shifts, among others. Despite the perceived threats of invasive species in sub-Saharan Africa, there are still limited capacities in dealing with this phenomenon, which may be attributed to existing knowledge gaps regarding the current status of alien invasive species and their control strategies [10]. To bridge these gaps, predictions of plant invasions in the African region, especially under global climate change, are very important to support development of policies and management programs for invasive species [9]. Exploring potential distributions of invasive species during temperature overshoot periods, i.e. periods with temperature increases of more than 1.5°C above the reference period (1986–2005) baseline in the course of the 21^st^ century, would help determine species survival boundary limits so as to guide management strategies. It is expected that warming temperatures of over 2°C above the reference period (1986–2005) baseline will exacerbate risks brought about by spread of invasive plant species if warming was maintained at 1.5°C and below [11]. To model the temperature rising scenarios, the Intergovernmental Panel on Climate Change (IPCC) 5^th^ Assessment Report [12] presents this in time-dependent projections of atmospheric greenhouse gas (GHG) concentrations in Representative Concentration Pathways (RCPs): the stringent mitigation scenario (RCP2.6) denoting a peak and decline of temperatures below 1.6°C, the intermediate stages (RCP4.5 and RCP6.0) denoting a stabilization without overshoot, and the higher GHG emission scenario (RCP8.5) denoting rising temperatures [9 p60, 12].

Risk assessments for invasive plant species range changes under changing climate can be done through building ecological niche models (ENMs) [13]. The approach taken by ENMs to determine potential species habitat changes is usually through deriving an empirical relationship between species distribution and abiotic factors such as climate [9] at various biogeographical scales [14–16] and then projecting the relationship to a given environment. Although projecting the models to the future time slice would require an accurate climate simulation for that given area, such site-specific climate models may not be available and instead bias-corrected global climate models are popularly used in ecological niche modeling studies with high levels of reliability [17]. The outputs from ENMs, usually risk maps, serve as baseline information for invasive species conservation management policy formulation [18, 19] so as to avert their spread and to apply appropriate eradication measures [13] as well as providing estimates of community-level species populations for immediate management prioritization, especially for well-established species [20].

There have been concerted efforts among researchers to document the status of invasive species in sub-Saharan Africa. Examples of existing work include that carried out by Witt *et al*. [6], whose output contributed greatly to the documentation of current status of invasive species occurring within the East African region. Among the species that were reported in their regional study included *Lantana camara* L., *Opuntia stricta* (Haw.) Haw, and *Acacia mearnsii* De Wild, which are all among the species listed in the land plant category of 100 worst invasive alien species [21]. Shackleton *et al*. [19] outlined that *L*. *camara* is a prevalent species in most parts of East Africa and has caused biodiversity and livelihood losses in some parts of Uganda while *O*. *stricta* has been found invasive mostly in arid and semi-arid areas of East Africa. In Laikipia County, a region in Kenya characterized by mostly rangeland land use, *O*. *stricta* has invaded about 17% of the area [22], and in the greater northern rangelands of Kenya, *Opuntia* spp. have been projected to increase in potential habitat range from ~183,000ha to ~206,900ha by the year 2070 [23]. In highland protected areas of Rwanda, *A*. *mearnsii* has survived as an understory species in pine and eucalyptus plantations and has generally done well in altitudes above 1200m including those outside natural forests [24]. Other species of interest that have been reported across the East African region include *Caesalpinia decapetala* (Roth) Alston, *Senna didymobotrya* (Fresen.) H.S. Irwin & Barneby and *Solanum campylacanthum* Hochst. ex A. Rich. *C*. *decapetala* is an alien species invading the East African highlands such as those in Kenya and Tanzania [25]. *S*. *campylacanthum* and *S*. *didymobotrya*, on the other hand, are both considered native species to tropical Africa and have been classified as among “problematic” species within the East Africa region due to their expansion in range [6]. They are both regarded as “bush encroachers” [26] or “extra-limital species” [6]. These terms are used to refer to native plant species which have become invasive. *S*. *didymobotrya* has been introduced elsewhere, e.g. in Australia and parts of America [26], for ornamental and medicinal purposes. It proliferates well in disturbed areas, along roadsides, riparian zones, bushland, savannas and wasteland habitats where it can form dense large stands, thereby resulting in displacement of native vegetation and restricting animal movement [25]. *S*. *campylacanthum* has widespread presence across East African countries in habitats such as roadsides, savanna grasslands, disturbed areas, and protected areas [25, 26]. Where the species has increased in density, other native species are displaced. In addition, its fruits are poisonous to grazing animals such as sheep and goats [25].

In Nyeri County, Kenya, where two of the most important biodiversity-rich ecosystems, i.e. Aberdare national park and the Mt. Kenya national park and forest ecosystem [27], as well as productive agricultural areas are located, there are limited studies on how climate change might affect the spread of invasive species in these habitats. For example, proliferation of invasive species such as *C*. *decapelata*, *S*. *campylacanthum*, *L*. *camara*, *Datura dothistroma*, *Resinus communis*, *Fraxinus pennsylvanica*, *A*. *mearnsii* and *Rubus steudneri* within the Mt. Kenya and Aberdare forest ecosystems has been reported by the Kenya Forest Service [28]. Inaction on management of invasive species may lead to costly habitat restoration efforts in invaded areas. For instance, uncontrolled spread of invasive species has already led to degradation of natural habitats in Kenya e.g. Mt. Marsabit forest, where the impact of invasive species has resulted in loss of forest cover from an initial 18,363 hectares in 1973 to 11,000 hectares by 2013 [29].

The objective of this study was therefore to explore different habitat suitability predictions for the periods 2050s and 2070s from different General Circulation Models (GCMs) and RCPs 2.6 (optimistic scenario), 4.5 (stable intermediate scenario) and 8.5 (pessimistic scenario) for the five studied invasive species in Kenya.

## Materials and methods

### Study area

**Fig 1:**
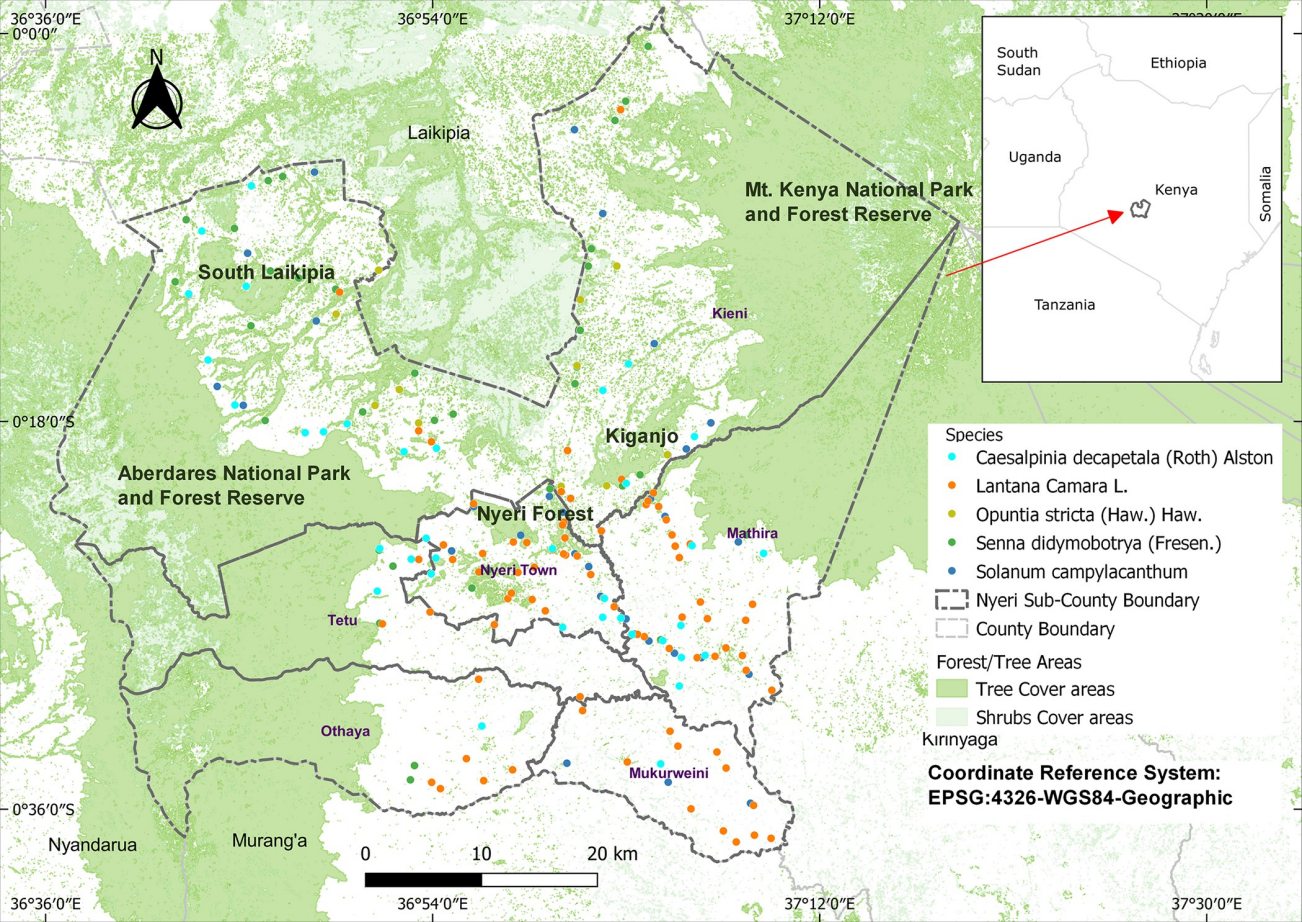
Study area map showing distribution of invasive species occurrence records. Data sources: (Administrative boundary layer: GADM database (www.gadm.org) under CC BY 4.0 license (https://gadm.org/license.html); Species presence data: GBIF.org (https://doi.org/10.15468/dl.v2peyj) and own roadside survey field work in Nyeri County under CC BY 4.0 license; Forest/tree cover layer: SERVIR GLOBAL data catalog [32] (https://servirglobal.net/Data-and-Maps) under CC BY 4.0 license.

This study was carried out in Nyeri County in central Kenya covering an area of ~ 3318 Km^2^. It is strategically located between Mt. Kenya ecosystem to the east and Aberdare ecosystem to the west within latitudes 0° 38’ 45” S and 0° 0’ 42” S and longitudes 36° 35’ 28” E and 37° 18’ 29” E (see protected areas in Fig 1). These two ecosystems and other isolated forest hills play a vital role in the climate of the area and serve as wildlife habitats, forest reserves and water catchment areas [30]. Nyeri County is comprised of Kieni, Othaya, Mathira, Mukurwe-ini, Tetu and Nyeri Town sub-counties (Fig 1). It has five agro-climatic zones: Humid (I), Sub humid (II), Semi humid (III), Semi humid to Semi-arid (IV) and Semi-Arid (V). Kieni sub-county falls in zones II, III, IV & V, Othaya, Nyeri Town and Tetu sub-counties fall in zones I & II, while Mathira and Mukurwe-ini sub-counties fall in zones I, II and III [31]. On average, annual rainfall in Nyeri ranges between 1200 – 1600mm and 500 – 1500mm during long and short rains, respectively, while the monthly mean temperatures range between 12–21˚C [30].

### Species occurrence records

Field survey records for five selected invasive species, namely *C*. *decapetala*, *L*. *camara*, *O*. *stricta*, *S*. *didymobotrya* and *S*. *campylacanthum*, were collected along selected road networks between October 2019 and February 2020 using a handheld GPS receiver (±3 m accuracy). Roadside surveys provide a cheaper alternative to estimate target species occurrence within a given area [33]. Furthermore, transport routes have been associated with introduction and eventual spread of invasive species [34, 35] and therefore act as a factor influencing dispersal [20]. Our target species also invade roadside habitats [25] and therefore probability of sighting them along survey routes was considered very high. Since forest edges provide favourable biotic and abiotic conditions for alien invasive species introduction and eventual spread to fragmented forest interiors [36], routes leading to forest edges were also considered.

As we drove along survey routes, target species occurrence locations were collected at approximately 2 to 5 km successive intervals. We selected a maximum of 5 km sampling interval to increase sampling intensity. The choice of interval was subjective as there are no reference standard intervals. For instance, Thapa *et al*. [37] and Henderson [38] used 5-10km while Wabuyele *et al*. [39] considered a 25km interval, indicating the subjective nature of the selection of intervals. A shorter interval of 2km was considered on routes with steeper terrain to account for vegetation diversity changes as elevation increases [37]. At each stop, species occurrence locations were collected on both sides of the road and in the adjacent habitats if any of the target species was sighted. The number of raw records collected within Nyeri County for individual species were as follows: 37 for *C*. *decapetala*, 141 for *L*. *camara*, 17 for *O*. *stricta*, 43 for *S*. *didymobotrya* and 72 for *S*. *campylacanthum*. Broennimann and Guisan [40] suggested fitting ENMs with a pool of data from a wide environmental range to avoid uncertainties when predicting species’ full invasion potential, more so in future climate scenarios. Therefore, to supplement our field data, we searched for existing presence records of the target species from GBIF.org [41]. We considered a pool of available presence data in selected East African regional countries, including Kenya, Ethiopia, Uganda, Rwanda, Burundi and Tanzania to capture the dominant environmental conditions in which the study species thrived. Regional models may perform better than continental or global models in capturing a species’ range margins [42]. The number of records that remained within the selected East African regional extent and the Nyeri County extent after rarefication were as shown in Table 1 and can be seen in S1 Appendix.

**Table 1:** Description of study species in terms of species scientific name and family and common name, life form, origin (adopted from Witt & Luke [25]) and the number of records used in ENM.

Species and Family name	Common name	Life form	Origin	Class	Species presence records (raw field data + GBIF data)	Rarefied records within the Selected East African region extent	Rarefied species data within Nyeri county extent
*Caesalpinia decapetala* (Roth) Alston (Fabaceae)	Mauritius or Mysore thorn	Evergreen shrub / climber	Native of Asia (India, Sri Lanka, China, Japan & Malaysia).	Alien	1384	1096	36
*Lantana camara* L. (Verbenaceae)	Lantana, tickberry	Tree or shrub	Subtropical and tropical America.	Alien	4467	3083	94
*Opuntia stricta* (Haw.) Haw. (Cactaceae)	Erect prickly pear	Succulent tree or shrub	South-east USA, eastern Mexico and some Caribbean Islands.	Alien	695	291	15
*Senna didymobotrya* (Fresen.) H.S. Irwin & Barneby (Fabaceae)	African senna	Tree or shrub	Tropical Africa	Native	1494	1101	44
*Solanum campylacanthum* Hochst. ex A. Rich. (Solanaceae)	Bitter apple, Sodom apple	Shrub	Africa, Middle East and India.	Native	2654	2113	72

### Preparation of presence and background data

#### Rarefying presence samples

We used the SDMtoolbox 2.4 for ArcGIS 10.5 [43] to spatially rarefy individual species occurrence data. Spatially clustered points introduce environmental biases and tend to affect a model’s ability to predict given new data [43]. We used 1 km Euclidean distance so as to match the 1 km^2^ spatial resolution of the predictor variables [44]. Respective records per species that were retained for model fitting are shown in Table 1.

#### Generating background data

Our species data lacked true absences and therefore background data or pseudo-absence data were generated randomly within the model fitting area for estimation of species-climate relationships. Barbet-Massin *et al*. [45] recommended the same number of pseudo-absences as available presences for machine learning methods and therefore, based on the number of rarefied species presence data (Table 1), a ratio of 1:1 for presence/pseudo-absence data for all the target species were used to fit models with the Random Forest (RF), Classification Tree Analysis (CTA) and Generalized Boosted Models (GBM) methods. On the other hand, the MAXENT method requires a large amount of background data as indicated by Phillips and Dudik [46], as it works by contrasting the environmental profile of the model fitting area against the environmental profile of available presence data and therefore background data was set to 10,000 for all the species. We also used 10,000 pseudo-absences for Generalized Additive Models (GAM) as suggested by Barbet-Massin *et al*. [45].

#### Data partitioning

Ecological niche models were calibrated and evaluated using all available species location data within the defined East African regional model fitting area (see S1 Appendix). We used the R package *“blockCV”* [47] to generate spatially separated training and testing folds to account for spatial autocorrelation inherent in species location data. Spatially separated blocks were generated using a specified size determined using the *“spatialAuto”* function provided in the package. The resulting blocks were then randomly assigned to five (5) cross-validation (cv) folds and set to output into a “*DataSplitTable*”, *biomod2* format, for subsequent use in a model fitting and evaluation cross-validation procedure. In this procedure, all species presence records and pseudo-absences are treated together within generated blocks and are subsequently assigned to the respective selected cv folds with allocation of locations to training or testing data [47]. Iterations for assigning of blocks to folds was set to 100 whereby the most evenly dispersed number of training and testing records was returned.

### Modelling predictor variables

We considered a set of predictor variables consisting of bioclimatic and topographic variables as described in Table 1 in S2 Appendix. The 19 standard WorldClim bioclimatic variables derived from averaged climate data for the period 1970–2000 [48] at a spatial resolution of 30 arc seconds (~1 km^2^) were downloaded from WorldClim (https://worldclim.org/). Digital Elevation Model (DEM) data at a spatial resolution of 12.5 x 12.5m were downloaded from the Alaska Satellite Facility [49]. Slope, aspect, plan and profile curvatures and topographic wetness index (twi) were derived from the DEM data. All predictor variable layers were resampled to 30 arc seconds spatial resolution and masked to the model fitting area extent (S1 Appendix) and to the Nyeri County extent (Fig 1).

Future climate data representing emission pathways for the period 2050s (2040–2069) and 2070s (2060–2089) [17] at 30 arc seconds spatial resolution were downloaded from (http://www.ccafs-climate.org/). We considered six Coupled Model Intercomparison Project Phase 5 (CMIP5) GCM data sets: *BCC*-CSM1.1(m), GFDL-*ESM2G*, *Hadgem2*-ES, *IPSL*-CM5A-MR, *MIROC*-ESM-CHEM, and *NCAR*-CCSM4 under the RCP2.6, RCP4.5 and RCP8.5. More information about these CMIP5 GCMs is presented in the work of Navarro-Racines *et al*. [17]. According to McSweeney *et al*. [50], these GCM models’ performance ratings on replicating timings for annual precipitation and temperature cycles are relatively similar with no significance differences in the Horn of Africa region within which our model calibration area lies.

### Predictor variables multi-collinearity analysis

A total of 25 model predictor variables (Table 1 in S2 Appendix) were subjected to multi-collinearity tests using Pearson’s correlation coefficient (r) and variance inflation factor (VIF). Calculation of VIF was as shown in Eq (1). Assessing and removing collinear variables conforms with statistical assumptions in regression models [51].

$$VIF=\frac{1}{\left(1-R_{j}^{2}\right)}$$ (1)

where $R_{j}^{2}$ is the coefficient of determination derived from model variables *j* [52].

We implemented this in R statistical software [53] and the ‘*usdm’* package [54]. We used the rarefied presence records for individual species to extract predictor values from all predictor variables and converted them into a matrix data frame for multi-collinearity testing. Uncorrelated predictor variables were identified through a stepwise procedure provided in the *‘vifcor’* function. The function finds a pair of predictor variables with maximum linear correlation (r > 0.70 [55]), and then excludes one of the variables having greater VIF value (VIF > 10 [56]). This procedure is repeated until the correlation coefficient between one remaining predictor variable and another is not greater than threshold. The remaining predictor variables were as shown in Table 2.

**Table 2:** Retained noncollinear predictor variables and their relative importance.

Species	*C*.* decapetala*	*L*.* camara*	*O*.* stricta*	*S*.* didymobotrya*	*S*.* campylacanthum*
**Explanatory Variables**	Aspect	Aspect	Aspect	Aspect	Aspect
	bio12	bio12	**bio12** ^a^	bio13	bio12
	bio13	**bio13** ^a^	bio14	**bio14** ^a^	bio13
	**bio14** ^a ^	**bio14** ^a^	**bio15** ^a^	bio18	**bio14** ^a^
	bio18	**bio15** ^a^	bio18	bio19	bio18
	bio19	bio18	bio19	**bio2** ^a^	bio19
	bio2	bio19	**bio4** ^a^	bio4	bio4
	bio4	bio2	bio7	bio5	**bio5** ^a^
	bio5	bio4	bio9	**Elevation** ^a^	bio7
	**Elevation** ^a^	bio5	Plan curvature	Plan curvature	Plan curvature
	Plan curvature	Elevation	Profile curvature	Profile curvature	Profile curvature
	Profile curvature	Plan curvature	Slope	Slope	Slope
	Slope	Profile curvature	twi	twi	twi
	twi	Slope			
		twi			

twi, topographic wetness index; bio2, Mean Diurnal Range (mean of monthly (max temp–min temp)); bio4, Temperature Seasonality (standard deviation × 100); bio5, Max Temperature of Warmest Month; bio7, Temperature Annual Range; bio9, Mean Temperature of Driest Quarter; bio12, Annual Precipitation; bio13, Precipitation of Wettest Month; bio14, Precipitation of Driest Month; bio15, Precipitation Seasonality—Coefficient of Variation; bio18, Precipitation of Warmest Quarter; bio19, Precipitation of Coldest Quarter

^**a**^Predictor variable with a relative variable importance of score *(1 –correlation) > 0*.*20* obtained in three or more ENM methods. Pearson correlation (*cor*) between model predictions obtained with shuffled predictor dataset and the reference dataset is used to obtain the predictor variable relative importance. Higher scores indicate a variable with high importance in a given model.

During the model fitting procedure, relative predictor variable importance was computed for analysis of the possible effects of changing climatic conditions on the study species. This was done through analysis of 2D plots of the ecological niche models’ response curves in the *biomod2* package [57]. In ecological niche studies, it is important to determine the relationship between a given bioclimatic predictor and the species response especially in light of changing climate conditions [58].

### Current and future species distribution modelling

#### Model fitting

ENMs were generated and evaluated using R statistical software [53] and the ‘*biomod2*’ package [59]. ENMs were built with 5 selected model fitting methods that are available in *biomod2*. Among these methods, the Random Forest (RF) algorithm has gained popularity in ecological niche modelling due to its good performance as reported in Zhang *et al*. [60] and by Shabani *et al*. [15] while the Maxent algorithm is popular due to its higher predictive power than traditional logistic regression [14] regardless of available species sample size [18]. The list of all methods used was as follows: one regression method (GAM, generalized additive models), three machine learning methods (Maxent [61]; RF, Random Forest; [62]; and GBM, generalized boosted models) and one classification method (CTA, classification tree analysis). Further details on the individual methods can be found in the work of Thuiller *et al*. [59]. All these methods are commonly used in ecological niche modelling as they use both species presence records and pseudo-absence/background data generated from noncollinear predictor variables [61, 63]. During model fitting, each presence observation and generated pseudo-absence point was assigned the same importance [57].

A data split table containing the training and testing records selected in the block cross-validation procedure was used for model training and model evaluation purposes. It should be noted that using all datasets may cause model overfitting and consequently fail to provide generality of the model for future time predictions [64]. To assess the predictor variables’ importance, we set the repetitions to 3 where the results were later used to obtain the average score per predictor variable. The other default settings for the individual model fitting methods were retained as provided in the *biomod2* package [57].

#### Model evaluation

Test data selected through the block cross-validation [47] technique were used for model accuracy evaluations. However, it should be noted that ENM predictive accuracy cannot be truly estimated due to lack of independent validation data [65]. Model evaluation metrics used were limited to those provided in the *biomod2* package. In *biomod2* some of the provided metrics include threshold-independent metrics such as the area under the receiver operating characteristics (ROC) curve (AUC) [66, 67] and the Boyce Index, which provides a reliable measure of presence-only methods for the fitted ENMs [68], and threshold-dependent metrics such as the True Skill Statistic (TSS) [69]. Although the ROC/AUC metric is often used as a measure of SDM model performance, it requires true presence-absence species data which is not usually available for building ENMs [15]. Readers are referred to work by Lobo *et al*. [70] discussing ROC/AUC applicability in model performance comparative analysis. They argued that the ROC/AUC metric should be avoided and pointed out the need to evaluate appropriate statistics to use for a given ENM application. Considering our study’s main aim, which was to predict environmentally suitable areas with available species presence data, a threshold-based metric, the TSS, was deemed appropriate to compare model performances and to transform continuous predictions to binary maps. As described by Allouche *et al*. [69], TSS combines both sensitivity and specificity of predictions to account for both commission and omission errors.

TSS values range from -1 to +1, where +1 indicates perfect agreement between observed values and predicted values while values of zero or less indicate a prediction no better than random chance [69]. Going by a classification of the model evaluation metric values outlined in the work of Zhang *et al*. [71], TSS values were interpreted as follows: < 0.4 were regarded as poor, 0.4–0.8 were regarded as moderate and > 0.8 were regarded as excellent.

The fitted species models were combined through ensemble modeling after excluding models with TSS values of < 0.6 for the study species *C*. *decapetala*, *L*. *camara*, *O*. *stricta*, and *S*. *didymobotrya*, while for *S*. *campylacanthum*, the threshold for model exclusions was set at < 0.5 following initial model result evaluations. The median probabilities algorithm was chosen to calculate the median values given by the models since it is less influenced by outliers [57]. In addition our species datasets were relatively large as we had considered all field and downloaded presence species records within the East African region as compared to the Nyeri County area [57]. Binary transformations were done using the cutoff value that maximizes the model’s accuracy according to the TSS statistics selected automatically within the *biomod2* model fitting procedure. This procedure was applied across all the chosen models for each of the retained model runs.

#### Model projections

Species potential habitats under current and future climate scenarios were projected across the Nyeri County geographical extent. Future species habitat suitability maps were produced using predictor variables of individual GCM (“individual GCM data”) as input to the fitted ecological niche models and using an ensemble of values for a given predictor variable, obtained by taking a simple average of corresponding projected values for that variable from the selected GCMs (“ensemble GCM data values”) prior to ecological niche modelling. As suggested in literature, the use of an ensemble of values from selected GCMs accounts for differences in climate predictions of individual GCM data [72, 73]. Individual plots of species range changes for each of the resulting species’ predictions from individual GCM data enabled visual exploration of the accrued prediction uncertainties. The function for building clamping masks for future predictions as provided in the *biomod2* package [57] was used to identify areas where predictions were uncertain due to out of range values that were not seen during model calibration. For easier comparison of the estimated clamping mask values, all the output masks values were scaled to a range of between 0–1. This information aimed at providing a basis for interpretation of the reliability of the models in predicting species potential habitat suitability.

#### Change distribution analysis

Species potential range change distribution analyses between the current and predicted future binary output maps were estimated using the *biomod2* package [59]. The relative number of pixels representing the potential habitats either gained, lost or stable for the prediction time periods were calculated and used for analysis of the expected species potential habitat turnover within our study area. To analyze differences in uncertainties from either taking a simple average of individual GCM data future predictions versus prediction from an ensemble of GCM data values as predictors, we presented the results from the two approaches in bar graphs to assess their relative levels of prediction uncertainty for our area of study. With reference to the current potential habitat suitability, the percentage gain and loss of habitat suitability was computed and the overall species habitat change computed by taking the difference between percentage gain minus percentage loss. Regarding the species’ ability to colonize new sites, it is well known that this kind of analysis would take into account factors such as the species’ dispersal ability to overcome geographical barriers and biotic interactions among co-occurring species [74]. We recognize that it is possible to model species potential spread through a user-defined time slice as provided in the “*MIGCLIM”* R package [75], where species dispersal simulations are done through defining parameters such as dispersal distances and kernels, geographical barriers, species propagule production and colonization of the predicted suitable habitat. However, there are limitations with defining reliable parameters, e.g. the respective species’ dispersal-related parameters such as the kernel distance, unless extensive field experiments for the study species have been conducted [75]. By the time this manuscript was completed, we had not come across such information to serve as parameters for our target species dispersal simulations in the *MIGCLIM* package. To simplify our simulations and respective analysis, we opted to go by two assumptions, namely the no dispersal and full/unlimited dispersal scenarios provided in the *biomod2* package [57].

## Results

### Ecological niche model performance

**Fig 2:**
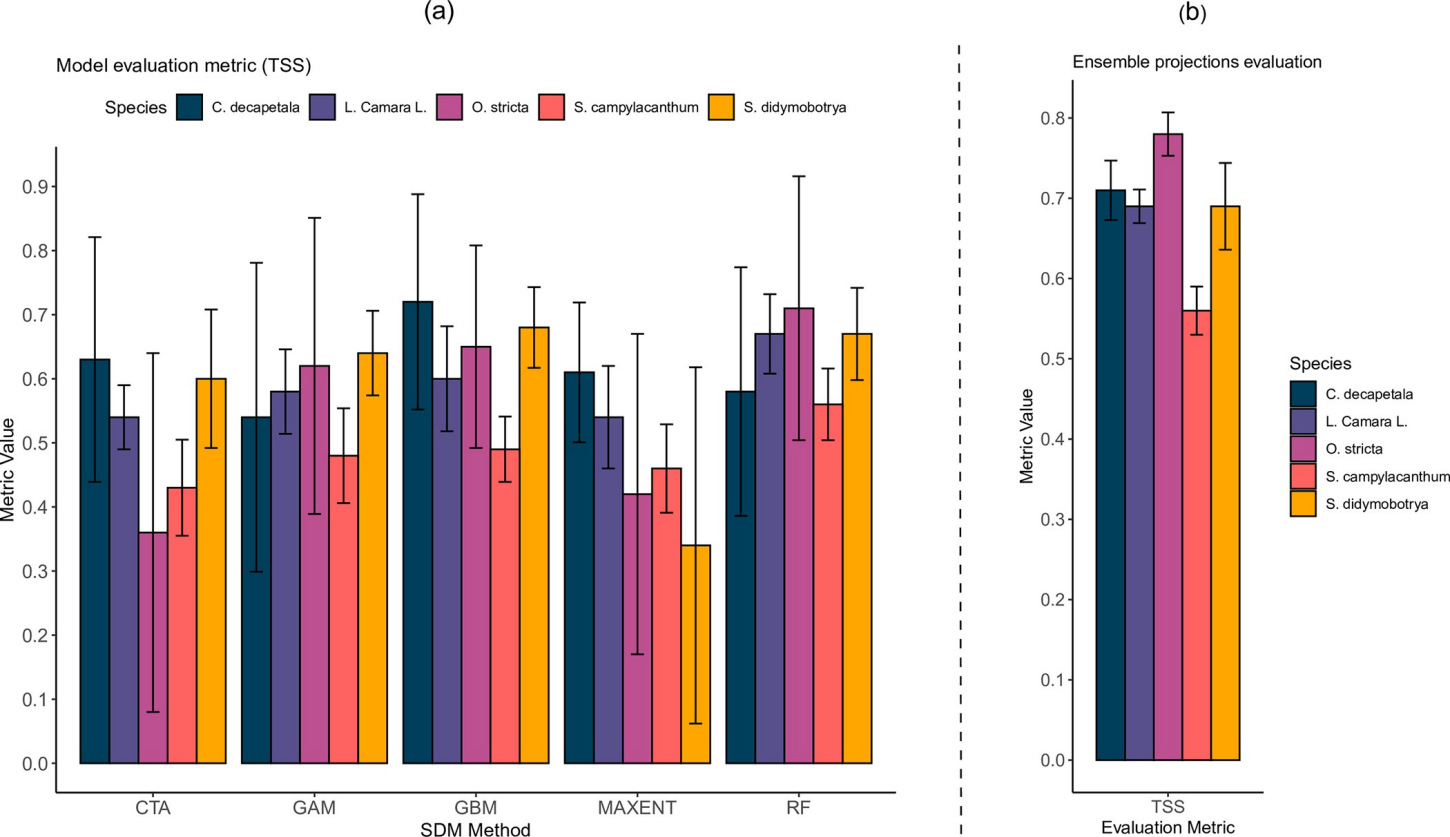
Bar graph plots of evaluation metric accuracies for the study species per ENM method and ensemble binary predictions.

Predictive performance of the fitted ENMs over the entire area was based on the TSS evaluation metric. Model performance per individual ENM method per individual species for the five folds cross-validated model runs and associated standard deviation is shown in Fig 2A. TSS evaluation metric values for *O*. *stricta* (0.36) and *S*. *didymobotrya* (0.34) obtained with the CTA and Maxent methods, respectively, were below useful values. However, model performances based on the TSS metric for the other species were above the “useful” value of ≥0.4. The highest TSS values were returned by the GBM method for *C*. *decapetala*.

Model performance can be influenced by the respective modelling method and the species being modelled. According to these model evaluation results, we find that for our target species, *C*. *decapetala* and *L*. *camara* were calibrated well with all the ENM methods. The worst results were obtained from CTA models for *O*. *stricta*. Maxent models performed poorly for *S*. *didymobotrya* as well. In summary, based on these metric results, RF, GBM and GAM methods can be regarded as the most reliable in terms of robustness of the built models for all of our study species.

Predictive performance of the ENMs in terms of the median ensemble binary predictions are shown in Fig 2B. The average values of the evaluation statistics from all the 5 cross-validated folds are indicated together with their standard deviations. The threshold-dependent values for TSS ranged between 0.56–0.78 (±*SD 0*.*021–0*.*054*). From these results, we see that there was moderate to very good discrimination of presences from absences for the resulting ensemble binary suitability maps of the study area.

### Relative importance of predictor variables

Results of relative predictor variable importance are summarized in Table 2 and a graphical representation per ENM method for individual species is shown in S3 Appendix. As per the criteria we applied (*relative variable importance with a score (1 –correlation) > 0*.*20 in 3 or more ENM methods*) to identify those that were relatively important during model fitting, not more than three explanatory variables were selected per ENM method for all study species. The variables selected as important varied among the species. They included: Annual Mean Diurnal Range (bio2), Temperature Seasonality (standard deviations) (bio4), Max Temperature of Warmest Month (bio5), Annual Precipitation (bio12), Precipitation of Wettest Month (bio13), Precipitation of Driest Month (bio14), Precipitation Seasonality (coefficient of variation) (bio15), and Elevation. Precipitation of Driest Month (bio14) was identified as important for *C*. *decapetala*, *L*. *camara*, *S*. *didymobotrya and S*. *campylacanthum*, an indication that these species are influenced by precipitation during the driest period of the year. The occurrence probabilities of these species’ increases in areas having precipitation values of ~12–25 mm during the driest month. The distribution of *L*. *camara* is affected in areas having more than 50% of monthly precipitation variability over the course of the year (bio15) while the response of *O*. *stricta* increases in areas with variability of between ~50 to ~60% followed by a decrease in response beyond ~60% of variability. As such, *O*. *stricta* can thrive well in areas with high annual temperature variations (above ~1.5°C of standard deviations–bio4) and in areas with relatively low annual precipitation (bio12) within values of between ~500–750 mm, while precipitation of wettest month (bio13) values of between ~100 – 300mm and above increases the probability of *L*. *camara* occurrence, signifying its ability to persist in high precipitation conditions during the year. *S*. *didymobotrya* shows a steady increase of probability of occurrence starting at ~9°C of mean of the monthly temperature ranges (bio2). Max Temperature of Warmest Month (bio5) values of between ~20–27°C increases the probability of occurrence of *S*. *campylacanthum* followed by gradual decrease in higher values up to ~40°C. High probability of occurrence of *C*. *decapetala* and *S*. *didymobotrya* species occurs in areas with elevation of between ~1000–2000 m above mean sea level and gradually decreases beyond 2000m above mean sea level.

### Current habitat suitability maps

**Fig 3:**
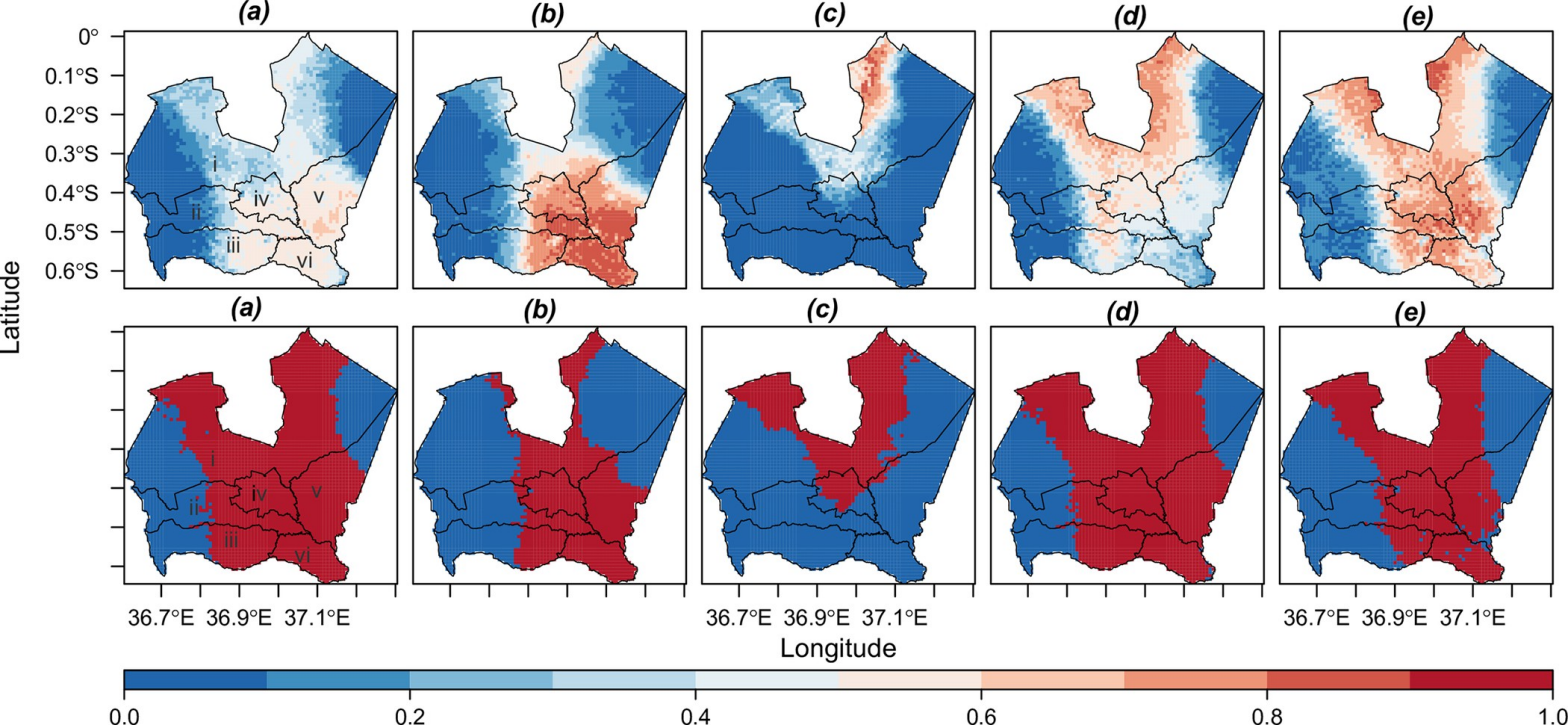
Species current potential ensemble probability suitability maps and respective median binary maps. Suitable areas on the scale are represented by red colour (1) while unsuitable areas are represented by blue colour (0). The black outline denotes the Nyeri sub-county boundaries labelled as follows: *i*, *Kieni; ii*, *Tetu; iii*, *Othaya; iv*, *Nyeri Town; v*, *Mathira; and vi*, *Mukurwe-ini Sub-counties and individual species as follows*: *(a)*, *C*. *decapetala (Roth) Alston; (b)*, *L*. *camara; (c)*, *O*. *stricta (Haw*.*) Haw*.*; (d)*, *S*. *didymobotrya; (e)*, *S*. *campylacanthum*. Data Source: (Administrative Boundary Layer: GADM database (www.gadm.org) under CC BY 4.0 license (https://gadm.org/license.html).

Projected binary species potential suitability maps showed that 67%, 40%, 28%, 68%, and 54% of the total study area (~ 3318 Km^2^) is suitable for the species *(a) C*. *decapetala*, *(b) L*. *camara*, *(c) O*. *stricta*, *(d) S*. *didymobotrya* and *(e) S*. *campylacanthum*, respectively (Fig 3). *O*. *stricta* had the least current suitable area among study species, occupying mostly parts of Nyeri Town, and in the semi-humid to semi-arid area of Kieni sub-county. *C*. *decapetala*, *S*. *didymobotrya*, and *S*. *campylacanthum* were predicted to have over 50% of the study area as suitable, mostly within the inhabited areas of all six sub-counties. *L*. *camara* suitable area covered mostly the southern parts of the study area and touched parts of all five of the sub-counties other than in Nyeri Town, where almost the whole area was predicted as suitable. Through visualization of the predicted current climate habitat suitability maps and an overlaid shapefile of forested areas, it was noted that potential species habitat occurred mostly on the periphery of the two major protected areas, i.e. the Mt. Kenya national reserve (areas to the north east of the study area) and the Aberdare national reserve (areas to the west of the study area), and within other fragmented forest areas around inhabited parts of the study area (see S4 Appendix).

### Future habitat suitability outputs

#### Model uncertainties

Future prediction maps are presented in S5 Appendix together with a measure of ensemble model extrapolation uncertainties, “clamping masks”. We visually analyzed the “clamping masks” based on the individual GCM data. The following notations were used to represent the full name of the GCM data: *bcc*, BCC-CSM1.1(m); *esm2g*, GFDL-ESM2G; *hadgem2*, Hadgem2-ES; *ipsl*, IPSL-CM5A-MR; *miroc*, MIROC-ESM-CHEM; and *ncar*, NCAR-CCSM4. Uncertainty in model predictions with all GCMs for *C*. *decapetala*, *L*. *camara*, and *S*. *didymobotrya* in the 2050s and 2070s were very low as compared to those obtained for *O*. *stricta* and *S*. *campylacanthum*. Notably, the *L*. *camara* model showed the lowest levels of extrapolation uncertainties among the study species with all the GCM climate data. This was an indication that future climate values provided by the GCM data were comparatively within the range of the values seen during model calibration of these species. A small section within Othaya sub-county had environmental values out of range for *S*. *didymobotrya* under *miroc* model data in all three RCP scenarios in both future time periods. Similarly, model predictions for *S*. *campylacanthum* with *bcc*, *esm2g* and *miroc* data under all RCPs in both future periods were uncertain in some areas, especially in the southeastern parts of Othaya and Tetu sub-counties and on the eastern side of Mathira sub-county.

Although future prediction maps for *O*. *stricta* were still generated despite the indication of model uncertainties in some areas as identified by the clamping masks, we observed that the species presence data were the fewest among the study species (Table 1 and Fig 1 in S1 Appendix) and perhaps inadequate to identify the complete environmental space of the species within our model calibration area as compared to the rest of the species. In summary, prediction models for *C*. *decapetala*, *L*. *camara*, and *S*. *didymobotrya* with *hadgem2*, *ipsl* and *ncar* GCM data had the lowest uncertainties while that of *O*. *stricta* was the highest.

#### Species future potential habitat changes

**Fig 4:**
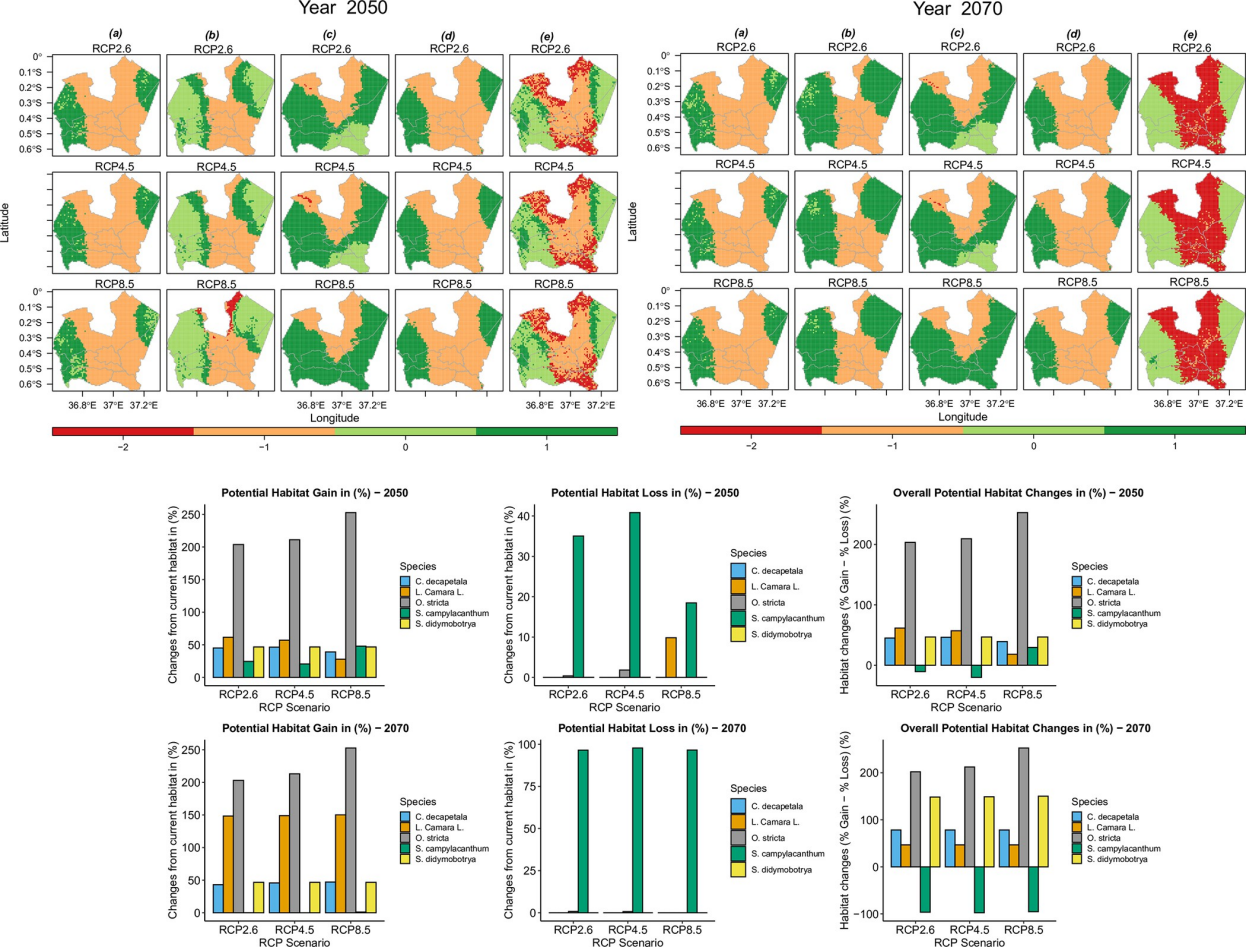
Predicted potential habitat gain, loss and overall habitat changes obtained from the predicted outputs from an average of bioclimatic variables of all GCM data. *-2 represents habitat loss*, *-1 represents suitable and stable in future*, *0 represents not suitable*, *1 represents habitat gain*. *Species labels are as follows*: *(a) L*. *camara; (b) C*. *decapetala (Roth) Alston; (c) O*. *stricta; (d) S*. *didymobotrya; (e) S*. *campylacanthum Hochst*. *ex A*. *Rich*.). Data Source: (Administrative Boundary Layer: GADM database (www.gadm.org) under CC BY 4.0 license (https://gadm.org/license.html).

The results for the potential habitat changes based on individual GCM data for the future periods 2050s and 2070s can be seen in Fig 1 in S6 Appendix, while those from the ensemble of the six GCM data values used as predictors are shown in Fig 4. Based on the simple average of outputs from individual GCMs (Fig 2 in S6 Appendix), assuming unlimited species dispersal, *C*. *decapetala* will have an overall habitat change (percentage gain minus percentage loss) of between ~ 44 to ~47% under the three RCP scenarios RCP2.6, RCP4.5 and RCP8.5. *L*. *camara* will have an overall habitat change of ~56 to ~59% under RCP2.6 and ~45% under RCPs 4.5 and 8.5 in both future periods. This indicated a slight decrease of the potential habitat gain under the RCP4.5 and RCP8.5 scenarios. *O*. *stricta* will have an overall habitat change of between ~203% to ~220% in both future periods, the highest among the study species. *S*. *didymobotrya* overall habitat change will remain the same at ~46% under all RCPs and in both future periods, an indication that changes in climatic conditions will have little effect on habitat suitability of the species within the area of study. *S*. *campylacanthum* will have a negative overall habitat change of ~ -23% to ~ -50% under RCP2.6 in the 2050s and RCP8.5 in the 2070s, respectively. This is an indication that the species’ potential habitat will be lost gradually as temperature conditions change under the three RCP scenarios. Results of the ensemble predictions differed slightly, especially for the 2070s period, from those obtained by taking a simple average of the six GCM outputs per RCP scenario. Nonetheless, the trends in the predicted results show that the transformed environmental conditions will bring about significant increase in potential suitable areas for *C*. *decapetala*, *L*. *camara*, *O*. *stricta*, and *S*. *didymobotrya* as opposed to a reduction of their potential suitable areas. *S*. *campylacanthum* is shown to be on a significant potential habitat decline by the year 2070.

#### Species dispersal

**Fig 5:**
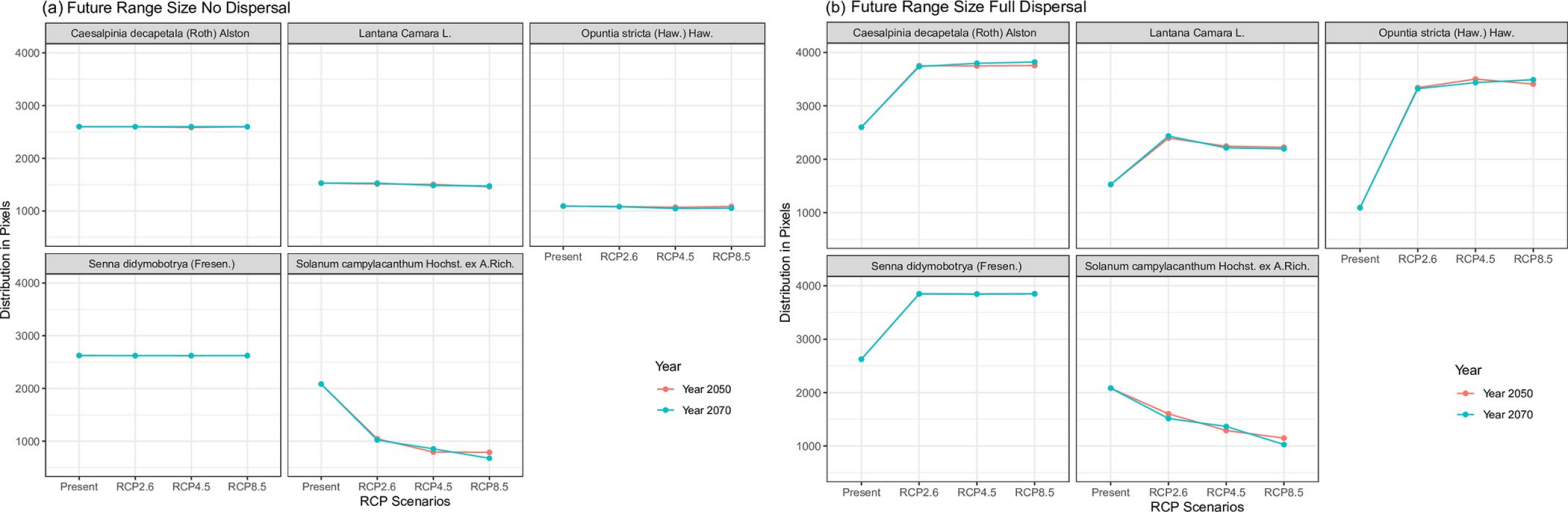
Predicted suitable areas under changing climate scenarios assuming no dispersal and full / unlimited dispersal of the species.

If there is no migration of the species (Fig 5A), which in this case is equated to the current occupied area that will remain occupied in the future, it was observed that, other than *S*. *campylacanthum*, which showed a decline of its current potential suitable areas, the rest of the species’ current potential habitats will largely be unaffected in the future environmental conditions. The trend seen with the assumption of unlimited species migration as shown in Fig 5B was that the *C*. *decapetala*, *L*. *camara*, *O*. *stricta and S*. *didymobotrya* potential habitat ranges will increase to new areas while *S*. *campylacanthum* will decrease in its predicted suitable areas.

## Discussion

Previous research has shown that climate change causes alterations to the current environmental parameters of a given area [76], which in turn causes expansion of invasive species’ suitable areas [72]. Our study shows that current predicted habitat for *C*. *decapetala*, *O*. *stricta*, and *S*. *didymobotrya* will persist and expand significantly to potential new habitats, followed closely by *L*. *camara*, whose suitable habitats will expand steadily but slowly into the surrounding uninhabited areas, especially into the Aberdare and Mt. Kenya national forest reserves. Instead of climate change enhancing range expansion, species may be subjected to an extinction trajectory. In our case, *S*. *campylacanthum* has the largest range contraction among the study species. Despite *S*. *campylacanthum* being a native species and presumably well adapted to its environment, the impacts of changing climatic conditions will likely exacerbate its suitable habitat decline and perhaps result in a possible shift to new environments outside our study area. On the contrary, distributions of alien invasive species such as *L*. *camara*, *C*. *decapetala*, and *O*. *stricta* will likely persist under these climatic changes. Our findings suggest that invasive species adapt well in diverse habitats under varying climatic conditions [8]. Although ENM outputs may give an underestimation or an overestimation of potential suitable areas of a given study species due to difficulties in predicting a species’ ability to evolve and adapt under changing conditions [76], our results suggest that climate change has a role to play in enhancing bio-invasions in natural environments [77].

Although changing climatic conditions will play a major role in reducing native species potential habitats in the future, continued decline and complete extinction is more likely to be influenced by habitat fragmentation over a longer period of time [78]. Land-use changes or habitat fragmentation influence species’ biological processes as much as climate change does [37, 76]. On the other hand, implying native species habitat decline as due to co-occurring alien invasive species may not suffice [79] unless a more practical framework such as a six-threshold framework outlined in the work of Downey and Richardson [78] is used to assess the role of alien plants in native plant extinctions. As indicated in the work of Bradley *et al*. [9], global increase in plant resources such as carbon dioxide (CO_2_) and nitrogen (N) favors the survival of invasive species and not native species whose survival depend on low nitrogen conditions in both disturbed and undisturbed areas. Daehler [80] also indicated that alien invasive species may perform relatively better than natives if there is high availability of light, nutrients or water resources and possibly due to inability of natives to produce adequate seeds to match that of invaders. On the other hand, most natives may persist under natural disturbance regimes, although there could be some alien invasive species which may outperform them in such regimes [80]. This means measuring the net effect of invasive species impacts on native species may require understanding the resource availability for a specific site, which in our case has not been quantified.

Contrary to perceived negative impacts in terms of habitat transformations by alien and native species as outlined in the work of Witt *et al*. [6], one of the listed problematic native species, *S*. *didymobotrya*, has benefits in African traditional medicine as demonstrated through research. Examples of such work on the species is Jeruto *et al*. [81], who found that its stem and root extracts have high efficiencies in inhibiting fungus growth, and Jeruto *et al*. [82], who found that the species’ root bark extracts possess phytochemical properties that inhibit bacterial pathogen growth. From a conservation point of view, making policies that aim at conserving *S*. *didymobotrya* especially in the wild would sustain availability of materials for development of such alternative sources of medicine. As we have established through this study, *S*. *didymobotrya* has a wide ecological niche as indicated by the future suitability maps which show potential habitat range covering the whole of Nyeri County including national protected areas. Owing to its importance in traditional medicine, more studies on the species’ current and future habitats for multiple counties should be prioritized to determine the absolute species distribution. As far as the other target study species are concerned, their negative impacts on natural habitats may outweigh their positive benefits. Many studies have advocated for urgent control measures for species such as *L*. *camara* due to its negative impacts on socio-economic livelihoods and biodiversity [2, 19]. This is because allelopathic action of *L*. *camara* individuals on co-occurring or neighboring plants [83] allows them to become better colonizers within introduced environments and enables their persistence where invasion has taken place [84]. In Nyeri County and the wider model calibration area (Table 1 and S1 Appendix), it is evident that *L*. *camara* is the most prominent species based on the number of presence records. *L*. *camara* proliferation, as observed during field data collection, is pronounced within isolated forest conservation areas such as the Muringato nursery, Nyeri municipality and Nyeri forest conservation areas, all of which fall within Nyeri Town sub-county (Fig 1) and mostly along land boundary hedges, road reserves and around homesteads. The species’ potential suitable habitat is predicted to persist through climate change scenarios, hence enabling the species to potentially migrate to neighboring uncolonized suitable areas, especially into the interior of the Aberdare and Mt. Kenya forest reserves. The populace in Nyeri County has continued to use *L*. *camara* as a suitable material for hedges around their parcel boundaries and homestead areas without knowing the dangers of the species to the natural environment. This kind of activity may be the leading cause of increased *L*. *camara* propagule production potential year-round and hence enhancing its dispersal by various agents to the uninhabited climatically suitable areas within the study area. The main vectors where these species occur and dispersal agents such as birds and other animals might determine the species’ dispersal patterns and distance, which in our case has not been assessed. For instance, in Kruger National Park in South Africa, rivers were identified as the main vectors by which *L*. *camara* generally travels, thereby informing on the spread dynamics of the species for better management [85].

Shackleton *et al*. [22] suggested urgent intervention measures on *O*. *stricta* in Laikipia County due to its impacts on annual economic losses per household among other negative impacts on rangelands. We foresee a similar scenario within our study area as the species’ potential habitat gain is the highest. In our study area, Kieni sub-county consists of mainly rangeland areas which are shown as potentially suitable. If the species’ propagules reach the Kieni sub-county rangelands, its invasion may affect pastoralism and wildlife conservation. A similar study by Ouko *et al*. [23] conducted within the northern rangeland areas including Nyeri County’s neighbor, Laikipia County, indicated a similar trend in potential habitat gain for this species, hence supporting the importance of performing habitat suitability in other areas. Although the number of presence records were limited for *O*. *stricta* and the resulting uncertainties reported in our results, the current study serves as a source of baseline information on the status of this particular species especially when prioritizing invasive species for management.

*S*. *didymobotrya*, a native plant species, shows good adaptability to warming climatic conditions. Its gain in habitat includes areas within the Mt. Kenya national park and forest reserve and the Aberdare national park and forest reserve (S4 and S6 Appendices). Extrapolation of a climate envelope [76] to the entire study area for the current climate shows predicted species suitable areas extend to the unsampled protected areas i.e. the Aberdare and Mt. Kenya national reserves shown in Fig 1 (S4 Appendix). Threats posed by alien plant invasion in high conservation areas are usually significant [78] and as observed from our future species habitat suitability maps, there will be continued proliferation of *C*. *decapetala*, *L*. *camara*, and *S*. *campylacanthum* within protected areas. These species were cited as a nuisance in these protected areas in a report by the Kenya Forest Service [28]. Although *S*. *campylacanthum* will likely experience a decline in suitable habitats in future environments, most of the habitat loss will be within the inhabited areas of the study area while habitat gains will be expected within the protected areas of the Aberdare and Mt. Kenya ecosystems (Fig 1e in S6 Appendix). However, this apparent range contraction may require a long time to take effect [76]. These results support the focus on *S*. *campylacanthum* which might continue being a nuisance within conservation areas. In a five-year research study by Pringle *et al*. [86] focusing on the effects of different sized mammalian herbivores including elephants, impala and dik-dik on *S*. *campylacanthum* populations within Mpala Research Centre in central Kenya, results indicated a complementary effect. It was indicated that, while dik-dik reduced much of the *S*. *campylacanthum* foliage, the impala and elephants contributed to seed dispersal hence complementing each other in sustaining the species’ population. Our study shows climate change will enhance the habitat range for *S*. *campylacanthum* in some parts of the Mt Kenya and Aberdare national reserves, and the species’ dispersal may be fast in these areas due to availability of seed dispersers. Referring to this case of *S*. *campylacanthum*, we infer that while ENMs may provide an understanding of the potential habitat dynamics of invasive species, studies on other drivers of species population distribution are equally important.

Ecological niche modeling depends on additional biotic factors (e.g. ability of species to compete for nutrients) and dispersal factors of a given species [74], which were not available to consider for our ENM predictions. Due to unavailability of such biotic and dispersal data, most researchers have used the readily available standard bioclimatic variables (mostly temperature and precipitation) as the only predictor variables for calibrating models for species future predictions, e.g. Ashraf *et al*. [87], especially in predicting risks of invasive species within a given region under changing climate conditions [9]. With increasing greenhouse gas emissions, it is expected that changes in global temperature and precipitation patterns will influence probability of occurrence of invasive species in a given area [9]. We noted that fewer bioclimatic variables were selected as important (Table 2) for our study species possibly because the calibration area had lower climate variation than perhaps that of a global spatial scale [51]. Other than bioclimatic variables, topographic variables such as elevation also play an important role in identifying conditions for species’ probability of occurrence [88]. For example, elevation was selected among the most important variables to determine species response for two of the study species, *C*. *decapetala and S*. *didymobotrya*, and therefore should not be ignored in ENM studies. Efforts geared towards determining the main driving factors for a given species’ response in a particular geographical area include analyzing the species’ responses to variations in precipitation values, temperature values and topographical attributes so as to provide information to conservation managers about areas meeting such conditions. We recognize that changes to species’ potential suitable habitat dynamics may be influenced by other factors not considered in the modeling process, such as changes of land use and land cover as well as nutrient deposition in soils, carbon dioxide levels [9] and the ability of the species to colonize newly predicted suitable climate space in consideration of dispersal limitations [75].

Future climate scenarios are captured through complex simulations referred to as General Circulation Models (GCMs) under different greenhouse gas emission pathways [74]. In our present study, we recognize that GCM data is a source of uncertainty in ENMs [72, 74]. From our initial selection of the “appropriate” GCM data for our model calibration region based on the findings of McSweeney *et al*. [50], our assessment of the ensemble model predictions using individual GCM data indicate different levels of model uncertainties in future habitat predictions and depending on a given species (S5 and S6 Appendices). Future predictions for *O*. *stricta* in all the selected GCM scenarios indicate higher model prediction uncertainties than any of the other study species. Therefore, further investigation is needed to determine its habitat suitability within the study area. Potential habitat changes presented in Fig 4 show that taking an average of the GCM data as ensemble predictors in future model predictions may not necessarily yield the same results as taking a simple average of individual GCM data model predictions (Fig 2 in S6 Appendix), hence indicating possible uncertainties related to climate data considered for modeling. However, a number of researchers have adopted the ensemble approach, e.g. [73], to account for the differences in GCM data. In our case, we adopted a simple average of the predicted outputs from the selected set of GCMs to quantify our species habitat range changes as opposed to the results from an ensemble of GCM data values. The rationale being, the differences were significant in absolute values and in predicted maps (see Figs 1 and 4 in S6 Appendix). Therefore, it is important to understand the effects of a given individual GCMs on future species predictions before taking an ensemble of GCMs data values for an ensemble of predictions.

In conservation planning, one of the main goals is to establish biogeographical patterns of a given species, often through ENMs. Such efforts enable identification of species invasiveness, sites that need prioritization for rehabilitation as well as re-introduction of threatened species [76]. The present study’s habitat suitability estimates for the current and future time periods serve as preliminary information for devising policies on effective management and monitoring actions in identified threatened habitats [51]. We have provided baseline information on potential distribution and range changes of study species within Nyeri County, a major milestone in moving towards maintaining healthy natural habitats and preventing climate change vulnerabilities such as the reduction of survival of endemic species and adverse effects on agricultural systems and water catchment areas brought about by uncontrolled spread of invasive species [89].

## Conclusions

Our study has provided baseline information on where and which invasive plant species will lose or gain suitable habitats and where the present climatically suitable areas will persist in future time periods. Immediate actions are needed to avert possible irreversible habitat transformation in predicted suitable areas especially for the habitat range expanding species *C*. *decapetala*, *L*. *camara*, *O*. *stricta* and *S*. *didymobotrya* if their migration reaches these areas. These invasive species are expected to remain a big threat to biodiversity hotspot areas especially in the Mt. Kenya and the Aberdare forest and national park reserves. The information generated through this study can be used to inform policy on prioritizing management of these species and determination of their absolute distributions within the area. These could be targeting small forest conservation areas or the entire geographical extent of the wider national reserve. Moving forward, we intend to improve on this work by developing a mapping framework that utilizes species’ unique spectral indices to enhance rapid estimation of fractional cover maps as well as derivation of essential biodiversity variables from remote sensing imagery for building subsequent species distribution maps.

## Supporting information

S1 Appendix Species geographical locations within the model fitting area. (PDF)

S2 Appendix Description of explanatory model predictor variables. (PDF)

S3 Appendix Predictor variable relative importance for the individual study species. (PDF)

S4 Appendix Forest outline layer overlaid on the current continuous and binary suitability maps. (PDF)

S5 Appendix Species future ensemble predictions and clamping masks for the future periods 2050s and 2070s. (PDF)

S6 Appendix Potential species habitat changes for the future periods 2050s and 2070s. (PDF)

S1 Dataset Rarefied field species presence records. (ZIP)

## References

[1] Richardson DM, Pysek P, Rejmanek M, Barbour MG, Panetta FD, West CJ. Naturalization and invasion of alien plants: concepts and definitions. Divers Distrib [Internet]. 2000 Mar;6(2):93–107. Available from: http://doi.wiley.com/10.1046/j.1472-4642.2000.00083.x

[2] Subhashni T, Lalit K. Impacts of climate change on invasive Lantana camara L. distribution in South Africa. African J Environ Sci Technol [Internet]. 2014;8(6):391–400. Available from: http://academicjournals.org/journal/AJEST/article-abstract/D9EF7A045968

[3] United Nations. United Nations, Transforming Our World: The 2030 Agenda for Sustainable Development [Internet]. 2015 [cited 2018 Oct 8]. Available from: https://sustainabledevelopment.un.org/content/documents/21252030 Agenda for Sustainable Development web.pdf

[4] Radosevich SR, Stubbs MM, Ghersa CM. Plant invasions-process and patterns. Weed Sci [Internet]. 2003;51:254–9. Available from: 10.1614/0043-1745(2003)051[0254:PIPAP]2.0.CO;2

[5] Royimani L, Mutanga O, Odindi J, Dube T, Matongera TN. Advancements in satellite remote sensing for mapping and monitoring of alien invasive plant species (AIPs). Phys Chem Earth, Parts A/B/C [Internet]. 2018 Dec [cited 2019 Jan 25]; Available from: https://linkinghub.elsevier.com/retrieve/pii/S1474706518301128

[6] Witt A, Beale T, van Wilgen BW. An assessment of the distribution and potential ecological impacts of invasive alien plant species in eastern Africa. Trans R Soc South Africa [Internet]. 2018 Sep 2;73(3):217–36. Available from: https://www.tandfonline.com/doi/full/10.1080/0035919X.2018.1529003

[7] Richardson DM, Pyšek P. What is an Invasive Species? [Internet]. Crop Protection Compendium. 2004 [cited 2020 Mar 6]. p. 17. Available from: https://www.cabi.org/isc/FullTextPDF/2009/20093238299.pdf

[8] Hellmann JJ, Byers JE, Bierwagen BG, Dukes JS. Five potential consequences of climate change for invasive species. Conserv Biol. 2008;22(3):534–43. doi: 10.1111/j.1523-1739.2008.00951.x 18577082

[9] Bradley BA, Blumenthal DM, Wilcove DS, Ziska LH. Predicting plant invasions in an era of global change. Trends Ecol Evol [Internet]. 2010;25(5):310–8. Available from: doi: 10.1016/j.tree.2009.12.003 20097441

[10] Early R, Bradley BA, Dukes JS, Lawler JJ, Olden JD, Blumenthal DM, et al. Global threats from invasive alien species in the twenty-first century and national response capacities. Nat Commun. 2016;7. doi: 10.1038/ncomms12485 27549569 PMC4996970

[11] IPCC, Allen M, Babiker M, Chen Y, de Coninck H, Connors S, et al. Summary for Policymakers. In: Global warming of 1.5°C. An IPCC Special Report. In 2018.

[12] IPCC. Climate Change 2014: Synthesis Report. Contribution of Working Groups I, II and III to the Fifth Assessment Report of the Intergovernmental Panel on Climate Change [Core Writing Team, R.K. Pachauri and L.A. Meyer (eds.)] [Internet]. 2014. Available from: ipcc.ch/site/assets/uploads/2018/05/SYR_AR5_FINAL_full_wcover.pdf

[13] Jiménez-Valverde A, Peterson AT, Soberón J, Overton JM, Aragón P, Lobo JM. Use of niche models in invasive species risk assessments. Biol Invasions. 2011;13(12):2785–97.

[14] Rivera ÓR de López-Quílez A. Development and Comparison of Species Distribution Models for Forest Inventories. ISPRS Int J Geo-Information [Internet]. 2017 Jun 16 [cited 2018 Dec 20];6(6):176. Available from: http://www.mdpi.com/2220-9964/6/6/176

[15] Shabani F, Kumar L, Ahmadi M. A comparison of absolute performance of different correlative and mechanistic species distribution models in an independent area. Ecol Evol. 2016;6(16):5973–86. doi: 10.1002/ece3.2332 27547370 PMC4983607

[16] Taylor S, Kumar L. Sensitivity Analysis of CLIMEX Parameters in Modelling Potential Distribution of Lantana camara L. PLoS One [Internet]. 2012 [cited 2018 Oct 8];7(7):40969. Available from: www.plosone.org doi: 10.1371/journal.pone.0040969 22815881 PMC3398004

[17] Navarro-Racines C, Tarapues J, Thornton P, Jarvis A, Ramirez-Villegas J. High-resolution and bias-corrected CMIP5 projections for climate change impact assessments. Sci Data [Internet]. 2020 Dec 20;7(1):7. Available from: http://www.nature.com/articles/s41597-019-0343-8 doi: 10.1038/s41597-019-0343-8 31959765 PMC6971081

[18] Truong TTA, Hardy GESJ, Andrew ME. Contemporary Remotely Sensed Data Products Refine Invasive Plants Risk Mapping in Data Poor Regions. Front Plant Sci [Internet]. 2017;8(May). Available from: http://journal.frontiersin.org/article/10.3389/fpls.2017.00770/full 10.3389/fpls.2017.00770 PMC5430062 28555147

[19] Shackleton RT, Witt ABR, Aool W, Pratt CF. Distribution of the invasive alien weed, Lantana camara, and its ecological and livelihood impacts in eastern Africa. African J Range Forage Sci. 2017;34(1):1–11.

[20] Brummer TJ, Maxwell BD, Higgs MD, Rew LJ. Implementing and interpreting local-scale invasive species distribution models. Franklin J, editor. Divers Distrib [Internet]. 2013 Aug;19(8):919–32. Available from: http://doi.wiley.com/10.1111/ddi.12043

[21] Lowe S, Browne M, Boudjelas S, Poorter M De. 100 of the World’s Worst Invasive Alien Species A selection from the Global Invasive Species Database. [Internet]. 2000. Available from: www.issg.org/booklet.pdf

[22] Shackleton RT, Witt ABR, Piroris FM, van Wilgen BW. Distribution and socio-ecological impacts of the invasive alien cactus Opuntia stricta in eastern Africa. Biol Invasions. 2017;19(8):2427–41.

[23] Ouko E, Omondi S, Mugo R, Wahome A, Kasera K, Nkurunziza E, et al. Modeling Invasive Plant Species in Kenya’s Northern Rangelands. Front Environ Sci. 2020;8(June).

[24] Seburanga JL. Black Wattle (Acacia mearnsii De Wild.) in Rwanda’s Forestry: Implications for Nature Conservation. J Sustain For. 2015;34(3):276–99.

[25] Witt A, Luke Q. Guide to the naturalized and invasive plants of Eastern Africa [Internet]. Witt A, Luke Q, editors. Wallingford, UK: CABI; 2017. vi + 601 pp. Available from: http://www.cabi.org/cabebooks/ebook/20173158959

[26] Lusweti A, Wabuyele E, Ssegawa P, Mauremootoo J. Invasive plants of East Africa (Kenya, Uganda and Tanzania), Lucid v. 3.5 key and fact sheets [Internet]. 2011 [cited 2020 Nov 25]. Available from: https://keys.lucidcentral.org/keys/v3/eafrinet/

[27] Government of the Republic of Kenya. Second Medium Term Plan, 2013–2017 [Internet]. Nairobi; 2013 [cited 2018 Oct 17]. Available from: http://vision2030.go.ke/inc/uploads/2018/06/Second-Medium-Term-Plan-2013-2017.pdf

[28] Kenya Forest Service. Aberdare forest reserve managment plan [Internet]. 2010 [cited 2020 Mar 4]. p. 94. Available from: http://www.kenyaforestservice.org/documents/Aberdare.pdf

[29] UNDP and County Government of Marsabit. Revised first county integrated development plan [Internet]. 2013. Available from: http://www.ke.undp.org/content/dam/kenya/docs/Democratic Governance/Marsabit County Revised CIDP.pdf

[30] Government of Kenya. Nyeri County Intergrated Development Plan 2018–2022 [Internet]. 2018. Available from: http://www.nyeri.go.ke/wp-content/uploads/2017/01/County-Govt-of-Nyeri-CIDP.pdf

[31] Gachathi F, Ngugi J, Omondi S. Useful trees suitable for central highlands eco-region [Internet]. Central Highlands Eco-region Research Programme, Kenya Forestry Research Institute (KEFRI); 2014 [cited 2020 Feb 3]. Available from: https://www.kefri.org/PDF/Leaflets/USEFULTREESSUITABLEFORCENTRALHIGHLANDSECO-REGION.pdf

[32] SERVIR GLOBAL. Kenya Sentinel2 Land Use Land Cover 2016 [Internet]. 2017. Available from: https://servirglobal.net/Data-and-Maps

[33] Shuster WD, Herms CP, Frey MN, Doohan DJ, Cardina J. Comparison of survey methods for an invasive plant at the subwatershed level. Biol Invasions. 2005;7(3):393–403.

[34] Meunier G, Lavoie C. Roads as Corridors for Invasive Plant Species: New Evidence from Smooth Bedstraw (Galium mollugo). Invasive Plant Sci Manag. 2012;5(1):92–100.

[35] Von Der Lippe M, Kowarik I. Long-distance dispersal of plants by vehicles as a driver of plant invasions. Conserv Biol. 2007;21(4):986–96. doi: 10.1111/j.1523-1739.2007.00722.x 17650249

[36] Dillon WW, Lieurance D, Hiatt DT, Clay K, Flory SL. Native and invasive woody species differentially respond to forest edges and forest successional age. Forests. 2018;9(7):1–17.

[37] Thapa S, Chitale V, Rijal SJ, Bisht N, Shrestha BB. Understanding the dynamics in distribution of invasive alien plant species under predicted climate change in Western Himalaya. Liu J, editor. PLoS One [Internet]. 2018 Apr 17;13(4):e0195752. Available from: https://dx.plos.org/10.1371/journal.pone.0195752 29664961 10.1371/journal.pone.0195752 PMC5903596

[38] Henderson L. Invasive, naturalized and casual alien plants in southern Africa: a summary based on the Southern African Plant Invaders Atlas (SAPIA). Bothalia [Internet]. 2007;37(2):215–48. Available from: http://abcjournal.org/index.php/ABC/article/view/322

[39] Wabuyele E, Lusweti A, Bisikwa J, Kyenune G, Clark K, Lotter WD, et al. A Roadside Survey of the Invasive Weed Parthenium hysterophorus (Asteraceae) in East Africa. J East African Nat Hist [Internet]. 2014;103(1):49–57. Available from: http://www.bioone.org/doi/10.2982/028.103.0105

[40] Broennimann O, Guisan A. Predicting current and future biological invasions: both native and invaded ranges matter. Biol Lett [Internet]. 2008 Oct 23;4(5):585–9. Available from: https://royalsocietypublishing.org/doi/10.1098/rsbl.2008.0254 18664415 10.1098/rsbl.2008.0254 PMC2610080

[41] GBIF.org. GBIF Occurrence Download [Internet]. [cited 2020 Dec 3]. Available from: 10.15468/dl.v2peyj

[42] Vale CG, Tarroso P, Brito JC. Predicting species distribution at range margins: testing the effects of study area extent, resolution and threshold selection in the Sahara-Sahel transition zone. Robertson M, editor. Divers Distrib [Internet]. 2014 Jan;20(1):20–33. Available from: https://onlinelibrary.wiley.com/doi/10.1111/ddi.12115

[43] Brown JL. SDMtoolbox: A python-based GIS toolkit for landscape genetic, biogeographic and species distribution model analyses. Methods Ecol Evol. 2014;5(7):694–700. 10.7717/peerj.4095 PMC5721907 29230356

[44] Elith J, Kearney M, Phillips S. The art of modelling range-shifting species. Methods Ecol Evol. 2010;1(4):330–42.

[45] Barbet-Massin M, Jiguet F, Albert CH, Thuiller W. Selecting pseudo-absences for species distribution models: How, where and how many? Methods Ecol Evol. 2012;3(2):327–38.

[46] Phillips SJ, Dudík M. Modeling of species distributions with Maxent: new extensions and a comprehensive evaluation. Ecography (Cop) [Internet]. 2008 Apr 1 [cited 2021 May 4];31(2):161–75. Available from: http://doi.wiley.com/10.1111/j.0906-7590.2008.5203.x

[47] Valavi R, Elith J, Lahoz‐Monfort JJ, Guillera‐Arroita G. blockCV: An r package for generating spatially or environmentally separated folds for k‐fold cross‐validation of species distribution models. Warton D, editor. Methods Ecol Evol [Internet]. 2019 Feb 8;10(2):225–32. Available from: https://onlinelibrary.wiley.com/doi/abs/10.1111/2041-210X.13107

[48] Fick SE, Hijmans RJ. Worldclim 2: New 1-km spatial resolution climate surfaces for global land areas. [Internet]. International Journal of Climatology. 2017 [cited 2019 Feb 2]. Available from: http://worldclim.org/version2

[49] Dataset ASF DAAC. ALOS PALSAR_Radiometric_Terrain_Corrected_high_res; Includes Material ©JAXA/METI [2007] [Internet]. 2007 [cited 2020 Jan 9]. Available from: 10.5067/Z97HFCNKR6VA

[50] McSweeney CF, Jones RG, Lee RW, Rowell DP. Selecting CMIP5 GCMs for downscaling over multiple regions. Clim Dyn [Internet]. 2015 Jun 16;44(11–12):3237–60. Available from: http://link.springer.com/10.1007/s00382-014-2418-8

[51] Manzoor SA, Griffiths G, Lukac M. Species distribution model transferability and model grain size—finer may not always be better. Sci Rep [Internet]. 2018;8(1):1–9. Available from: 10.1038/s41598-018-25437-1 PMC5940916 29740002

[52] Jackson LS, Carslaw N, Carslaw DC, Emmerson KM. Modelling trends in OH radical concentrations using generalized additive models. Atmos Chem Phys. 2009;9(6):2021–33.

[53] R Core Team. R: A Language and Environment for Statistical Computing [Internet]. Vienna, Austria: R Foundation for Statistical Computing; 2021. Available from: http://www.r-project.org/

[54] Naimi B. Package “usdm”. Uncertainty Analysis for Species Distribution Models. R- Cran. 2017;

[55] van Proosdij ASJ, Sosef MSM, Wieringa JJ, Raes N. Minimum required number of specimen records to develop accurate species distribution models. Ecography (Cop) [Internet]. 2016 Jun;39(6):542–52. Available from: http://doi.wiley.com/10.1111/ecog.01509

[56] Naimi B, Araújo MB. sdm: a reproducible and extensible R platform for species distribution modelling. Ecography (Cop) [Internet]. 2016 Apr;39(4):368–75. Available from: http://doi.wiley.com/10.1111/ecog.01881

[57] Thuiller W, Georges D, Engler R, Breiner F. biomod2: Ensemble Platform for Species Distribution Modeling [Internet]. 2020. Available from: https://cran.r-project.org/web/packages/biomod2/index.html

[58] O’Donnell MS, Ignizio DA. Bioclimatic Predictors for Supporting Ecological Applications in the Conterminous United States [Internet]. U.S. Geological Survey Data Series 691, Reston, Virginia; 2012 [cited 2021 May 5]. Available from: https://pubs.er.usgs.gov/

[59] Thuiller W, Lafourcade B, Engler R, Araújo MB. BIOMOD—A platform for ensemble forecasting of species distributions. Ecography (Cop). 2009;32(3):369–73.

[60] Zhang L, Huettmann F, Liu S, Sun P, Yu Z, Zhang X, et al. Classification and regression with random forests as a standard method for presence-only data SDMs: A future conservation example using China tree species. Ecol Inform [Internet]. 2019;52(1):46–56. Available from: 10.1016/j.ecoinf.2019.05.003

[61] Phillips SJ, Anderson RP, Schapire RE. Maximum entropy modeling of species geographic distributions. Ecol Modell [Internet]. 2006 [cited 2019 Feb 2];190:231–59. Available from: http://webpages.icav.up.pt/ptdc/BIA-BIC/110587/2009/Papers/20.pdf

[62] Breiman L. Random Forests. Mach Learn [Internet]. 2001;45(1):5–32. Available from: http://link.springer.com/10.1023/A:1010933404324

[63] Hao T, Elith J, Guillera‐Arroita G, Lahoz‐Monfort JJ. A review of evidence about use and performance of species distribution modelling ensembles like BIOMOD. Serra‐Diaz J, editor. Divers Distrib [Internet]. 2019 May 22 [cited 2021 May 4];25(5):839–52. Available from: https://onlinelibrary.wiley.com/doi/abs/10.1111/ddi.12892

[64] Radosavljevic A, Anderson RP. Making better Maxent models of species distributions: Complexity, overfitting and evaluation. J Biogeogr. 2014;41(4):629–43.

[65] Barbet-Massin M, Rome Q, Villemant C, Courchamp F. Can species distribution models really predict the expansion of invasive species? PLoS One. 2018;13(3):1–14. doi: 10.1371/journal.pone.0193085 29509789 PMC5839551

[66] Pearce J, Ferrier S. Evaluating the predictive performance of habitat models developed using logistic regression. Ecol Modell. 2000;133(3):225–45.

[67] McPherson JM, Jetz W, Rogers DJ. The effects of species’ range sizes on the accuracy of distribution models: ecological phenomenon or statistical artefact? J Appl Ecol [Internet]. 2004 Sep 30;41(5):811–23. Available from: http://doi.wiley.com/10.1111/j.0021-8901.2004.00943.x

[68] Hirzel AH, Le Lay G, Helfer V, Randin C, Guisan A. Evaluating the ability of habitat suitability models to predict species presences. Ecol Modell [Internet]. 2006 Nov;199(2):142–52. Available from: https://linkinghub.elsevier.com/retrieve/pii/S0304380006002468

[69] Allouche O, Tsoar A, Kadmon R. Assessing the accuracy of species distribution models: Prevalence, kappa and the true skill statistic (TSS). J Appl Ecol. 2006;43(6):1223–32.

[70] Lobo JM, Jiménez-Valverde A, Real R. AUC: a misleading measure of the performance of predictive distribution models. Glob Ecol Biogeogr [Internet]. 2008 Mar;17(2):145–51. Available from: https://onlinelibrary.wiley.com/doi/10.1111/j.1466-8238.2007.00358.x

[71] Zhang L, Liu S, Sun P, Wang T, Wang G, Zhang X, et al. Consensus forecasting of species distributions: The effects of niche model performance and niche properties. PLoS One. 2015;10(3). doi: 10.1371/journal.pone.0120056 25786217 PMC4364626

[72] Shrestha UB, Shrestha BB. Climate change amplifies plant invasion hotspots in Nepal. Vaclavik T, editor. Divers Distrib [Internet]. 2019 Oct 2;25(10):1599–612. Available from: https://onlinelibrary.wiley.com/doi/abs/10.1111/ddi.12963

[73] Aguirre-Gutiérrez J, van Treuren R, Hoekstra R, van Hintum TJL. Crop wild relatives range shifts and conservation in Europe under climate change. Divers Distrib. 2017;23(7):739–50.

[74] Pearson RG. Species’ Distribution Modeling for Conservation Educators and Practitioners. Lessons Conserv [Internet]. 2007;3:54–89. Available from: http://ncep.amnh.org/linc

[75] Engler R, Hordijk W, Guisan A. The MIGCLIM R package—seamless integration of dispersal constraints into projections of species distribution models. Ecography (Cop) [Internet]. 2012 Oct;35(10):872–8. Available from: http://doi.wiley.com/10.1111/j.1600-0587.2012.07608.x

[76] Sinclair SJ, White MD, Newell GR. How useful are species distribution models for managing biodiversity under future climates? Ecol Soc. 2010;15(1).

[77] Shrestha UB, Sharma KP, Devkota A, Siwakoti M, Shrestha BB. Potential impact of climate change on the distribution of six invasive alien plants in Nepal. Ecol Indic. 2018;95:99–107.

[78] Downey PO, Richardson DM. Alien plant invasions and native plant extinctions: a six-threshold framework. AoB Plants. 2016;8:plw047. doi: 10.1093/aobpla/plw047 27422543 PMC4972473

[79] Gurevitch J, Padilla DK. Are invasive species a major cause of extinctions? Trends Ecol Evol. 2004;19(9):470–4. doi: 10.1016/j.tree.2004.07.005 16701309

[80] Daehler CC. Performance Comparisons of Co-Occurring Native and Alien Invasive Plants: Implications for Conservation and Restoration. Annu Rev Ecol Evol Syst [Internet]. 2003 Jul 9;34:183–211. Available from: http://www.jstor.org/stable/30033774

[81] Jeruto P, Arama PF, Anyango B, Akenga T, Nyunja R, Khasabuli D. In vitro antifungal activity of methanolic extracts of different senna didymobotrya (fresen.) H.S. Irwin & barneby plant parts. African J Tradit Complement Altern Med. 2016; 10.21010/ajtcam.v13i6.24 PMC5412189 28480375

[82] Jeruto P, Arama PF, Anyango B, Maroa G. Phytochemical screening and antibacterial investigations of crude methanol extracts of Senna didymobotrya (Fresen.) H. S. Irwin & Barneby. J Appl Biosci [Internet]. 2017 Sep 27;114(1):11357. Available from: https://www.ajol.info/index.php/jab/article/view/161557

[83] Sharma OP, Sharma S, Pattabhi V, Mahato SB, Sharma PD. A review of the hepatotoxic plant Lantana camara. Crit Rev Toxicol. 2007;37(4):313–52. doi: 10.1080/10408440601177863 17453937

[84] Priyanka N, Joshi PK. A review of Lantana camara studies in India. Int J Sci Res Publ [Internet]. 2013 [cited 2018 Oct 8];3(10). Available from: www.ijsrp.org

[85] Vardien W, Richardson DM, Foxcroft LC, Thompson GD, Wilson JRU, Le Roux JJ. Invasion dynamics of Lantana camara L. (sensu lato) in South Africa. South African J Bot [Internet]. 2012;81:81–94. Available from: 10.1016/j.sajb.2012.06.002

[86] Pringle RM, Goheen JR, Palmer TM, Charles GK, DeFranco E, Hohbein R, et al. Low functional redundancy among mammalian browsers in regulating an encroaching shrub (Solanum campylacanthum) in African savannah. Proc R Soc B Biol Sci. 2014; 10.1098/rspb.2014.0390 PMC4024297 24789900

[87] Ashraf U, Peterson AT, Chaudhry MN, Ashraf I, Saqib Z, Ahmad SR, et al. Ecological niche model comparison under different climate scenarios: A case study of Olea spp. in Asia. Ecosphere. 2017;8(5):1–13.29552374

[88] Radosevich SR, Stubbs MM, Ghersa CM. Plant invasions—process and patterns. Weed Sci [Internet]. 2003 [cited 2022 Feb 10];51(2):254–9. Available from: https://www.cambridge.org/core/product/34BAD7D3C3AD0CA8CC14348C991589A9

[89] IUCN. Invasive alien species and climate change [Internet]. 2017 [cited 2019 Feb 2]. Available from: https://www.iucn.org/sites/dev/files/ias_and_climate_change_issues_brief_final.pdf

