# Supplementary material for: Assessing distribution changes of selected native and alien invasive plant species under changing climatic conditions in Nyeri County, Kenya
Source: PLoS One. 2022 Oct 3;17(10):e0275360. doi: 10.1371/journal.pone.0275360 (PMC9529121; doi:10.1371/journal.pone.0275360)

S1 Appendix. Species geographical locations within the model fitting area.

Fig 1. Geographical distribution of study species occurrence data used in fitting ecological niche models. The selected east African regional countries consist of Kenya, Ethiopia, Uganda, Rwanda, Burundi and Tanzania. *(a) L. camara; (b) C. decapetala (Roth) Alston; (c) O. stricta; (d) S. didymobotrya; (e) S. campylacanthum Hochst. ex A. Rich.*

Data Sources:

Map layout and design produced by Author showing Administrative Boundary Layer obtained from GADM database (www.gadm.org) under CC BY 4.0 license (https://gadm.org/license.html); and Species Presence Data obtained from GBIF.org (https://doi.org/10.15468/dl.v2peyj) and Roadside survey field work done by author in Nyeri county under CC BY 4.0 license.

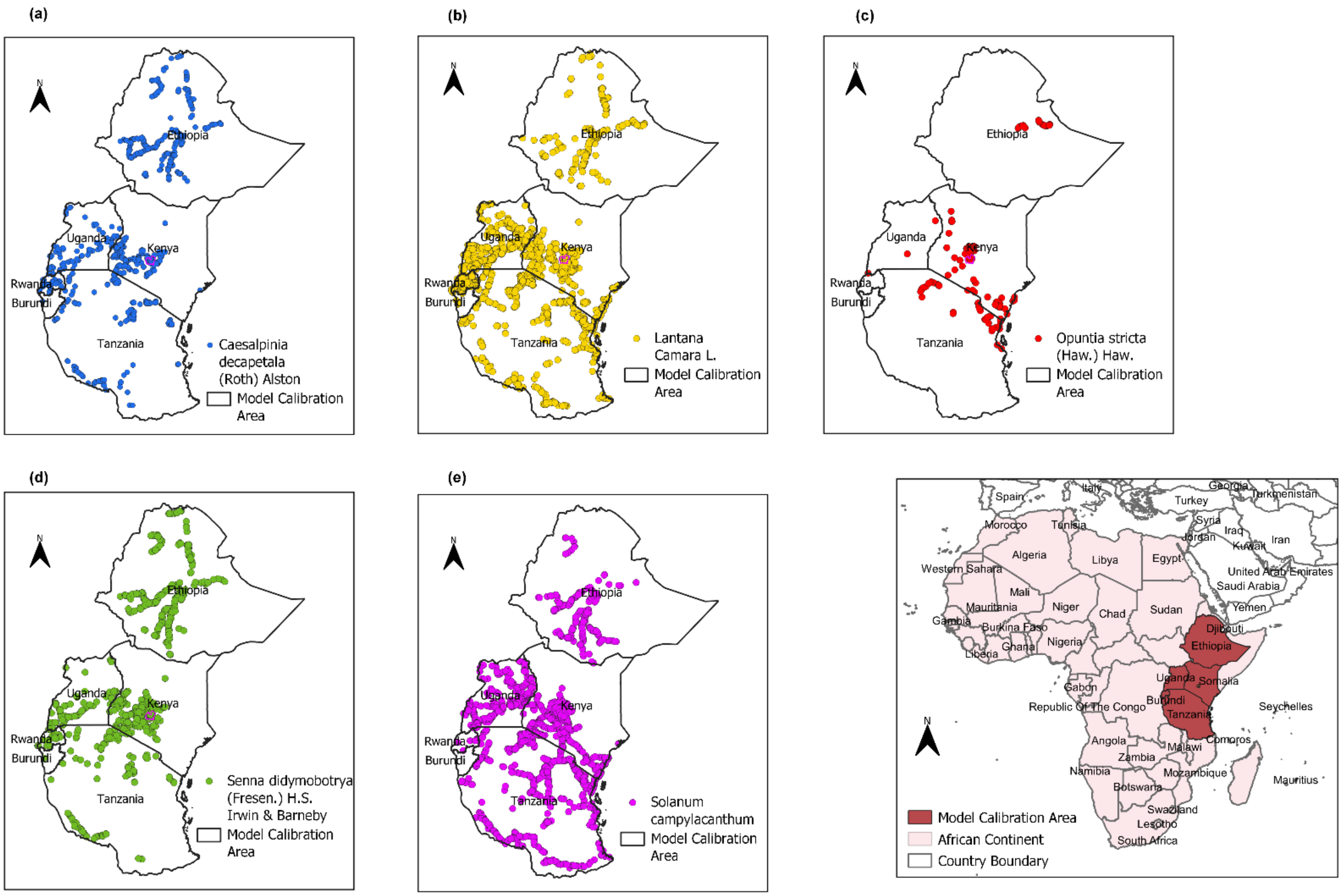

Supplement: S1 Appendix — (PDF) [file pone.0275360.s001.pdf]
