# Supplementary material for: Assessing distribution changes of selected native and alien invasive plant species under changing climatic conditions in Nyeri County, Kenya
Source: PLoS One. 2022 Oct 3;17(10):e0275360. doi: 10.1371/journal.pone.0275360 (PMC9529121; doi:10.1371/journal.pone.0275360)
Supplement: S2 Appendix — (PDF) [file pone.0275360.s002.pdf]

S2 Appendix. Description of explanatory model predictor variables.

Table 1. Description of model predictor variables considered from literature review from [1–3].

Variable	Type	Ecological importance	Description	Source
19 standard bioclimatic variables	Abiotic/Environment	Provide information on climatic variables (temperature and precipitation) necessary for plants growth.	30arc sec grid (~ 1km at the equator), Worldclim version 2 data	[4]
Digital Elevation Model (DEM)	Abiotic/Environment	Elevation values indicate topographic relief i.e. heights of surface points relative to a vertical datum frame.	Original DEM spatial resolution was 12.5m x 12.5m. All these layers were resampled to 30 arc seconds or ~ 1km at the equator to match WorldClim version 2 data.	[5]
Slope	Abiotic/Environment	Signifies the rate of maximum change vertically across surfaces		Derived from DEM
Aspect	Abiotic/Environment	Defines the direction of flow of water across surfaces. Shows the slope direction.		Derived from DEM
plan and profile curvatures	Abiotic/Environment	Affects the behavior of water flow with respect to speed across surfaces.		Derived from DEM
Topographic wetness index (TWI))	Abiotic/Environment	Indicates soil moisture distribution across surfaces [6]. High values indicate potential water accumulation areas.		Derived from DEM

References

1. Truong TTA, Hardy GESJ, Andrew ME. Contemporary Remotely Sensed Data Products Refine Invasive Plants Risk Mapping in Data Poor Regions. Front Plant Sci [Internet]. 2017;8(May). Available from: http://journal.frontiersin.org/article/10.3389/fpls.2017.00770/full

2. Rahimian Boogar A, Salehi H, Pourghasemi HR, Blaschke T. Predicting Habitat Suitability and Conserving Juniperus spp. Habitat Using SVM and Maximum Entropy Machine Learning Techniques. Water. 2019;11(10):2049.

3. Qin Z, Zhang JE, DiTommaso A, Wang RL, Liang KM. Predicting the potential distribution of Lantana camara L. under RCP scenarios using ISI-MIP models. Clim Change. 2016;134(1–2):193–208.

4. Fick SE, Hijmans RJ. Worldclim 2: New 1-km spatial resolution climate surfaces for global land areas. [Internet]. International Journal of Climatology. 2017 [cited 2019 Feb 2]. Available from: http://worldclim.org/version2

5. Dataset ASF DAAC. ALOS PALSAR_Radiometric_Terrain_Corrected_high_res; Includes Material ©JAXA/METI [2007] [Internet]. 2007 [cited 2020 Jan 9]. Available from: https://doi.org/10.5067/Z97HFCNKR6VA

6. Raduła MW, Szymura TH, Szymura M. Topographic wetness index explains soil moisture better than bioindication with Ellenberg's indicator values. Ecol Indic. 2018;85(October 2017):172–9.
