# Supplementary material for: Assessing distribution changes of selected native and alien invasive plant species under changing climatic conditions in Nyeri County, Kenya
Source: PLoS One. 2022 Oct 3;17(10):e0275360. doi: 10.1371/journal.pone.0275360 (PMC9529121; doi:10.1371/journal.pone.0275360)

S3 Appendix. Relative Importance of predictor variables for individual study species.

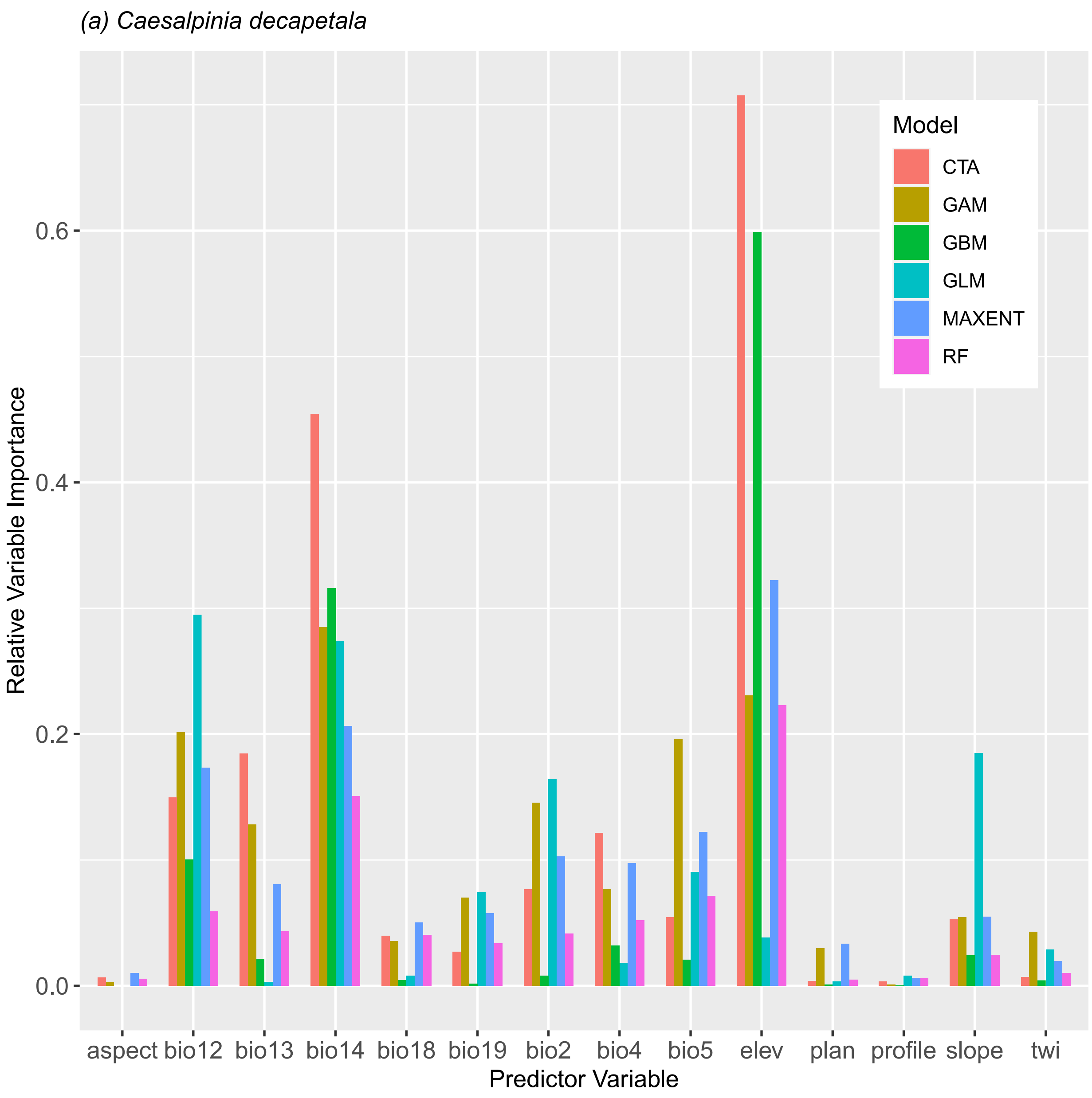

(b) Lantana Camara

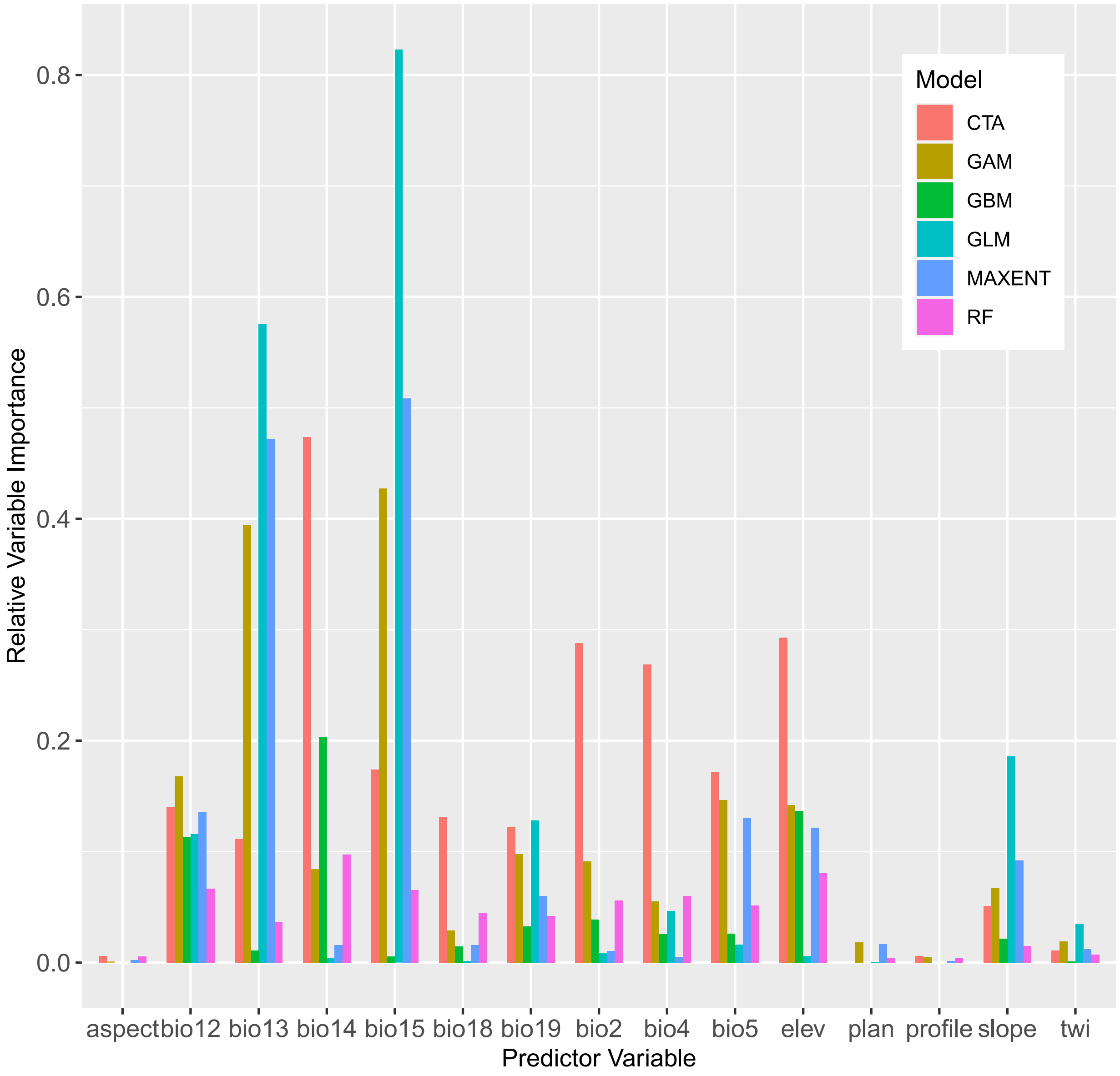

(c) opuntia stricta

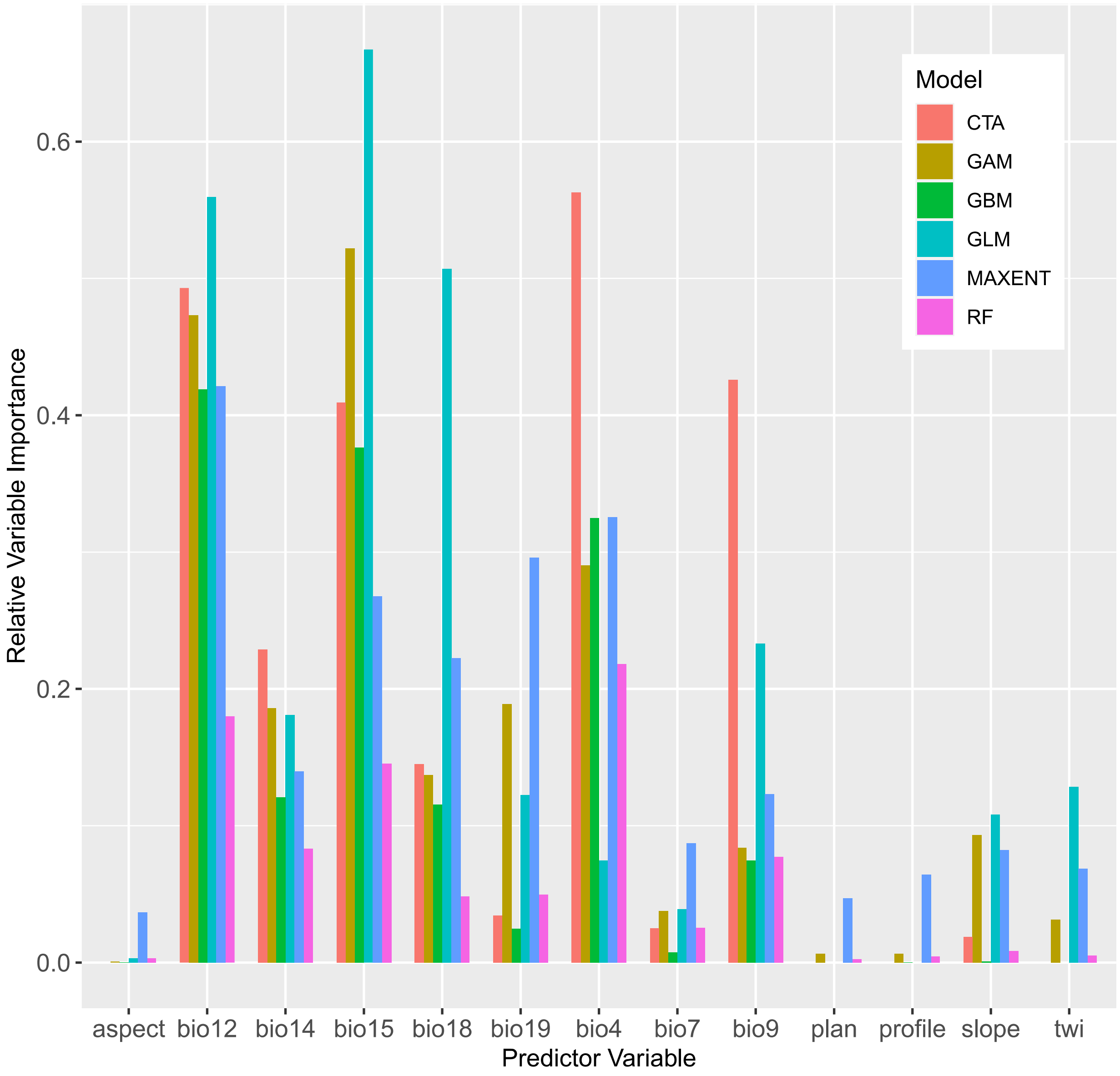

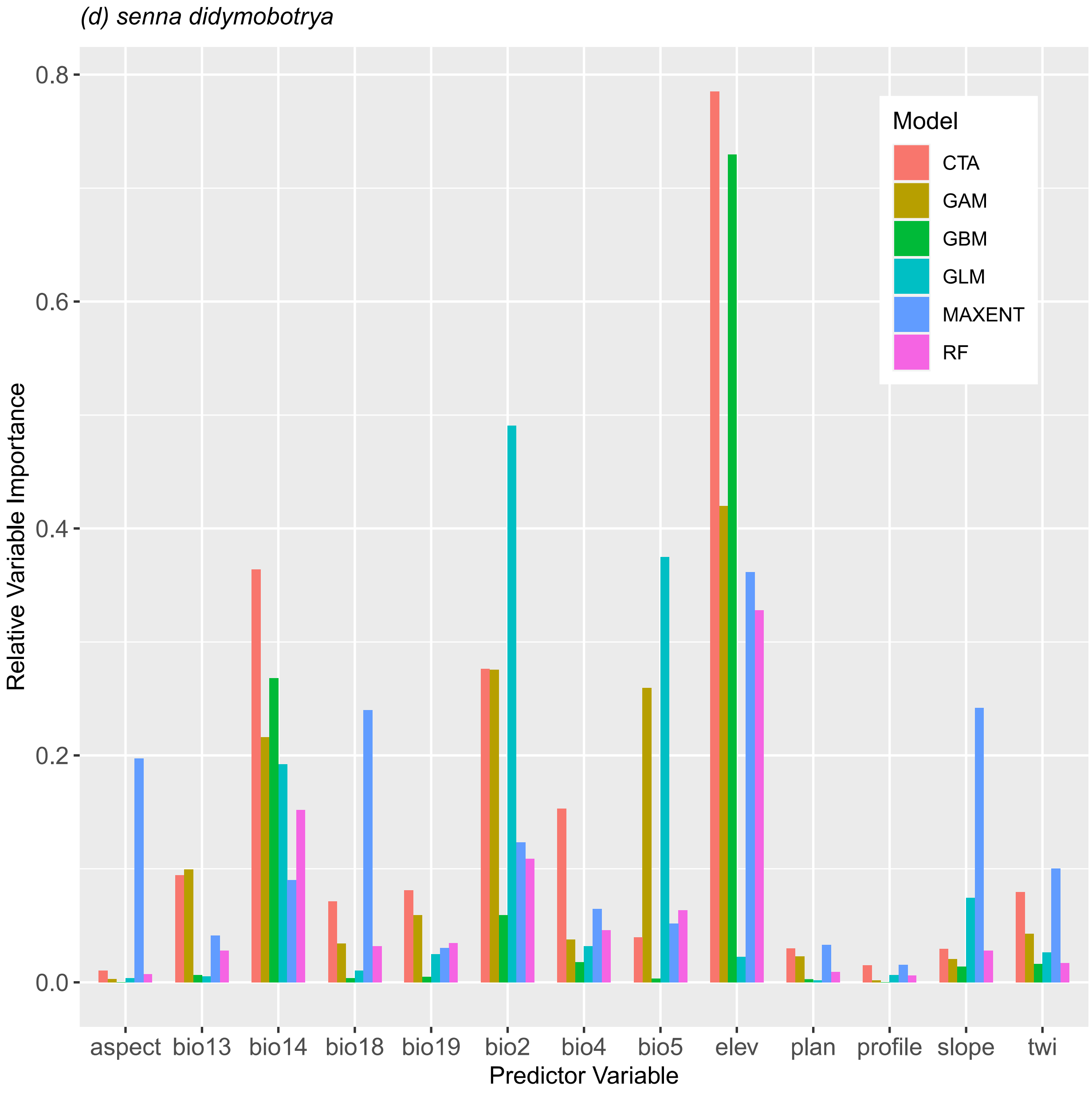

(d) senna didymobotrya
Relative Variable Importance
0.8
0.6
0.4
0.2
0.0
Model
CTA
GAM
GBM
GLM
MAXENT
RF
aspect
bio13
bio14
bio18
bio19
bio2
bio4
bio5
elev
plan
profile
slope
twi
Predictor Variable

(e) solanum campylacanthum

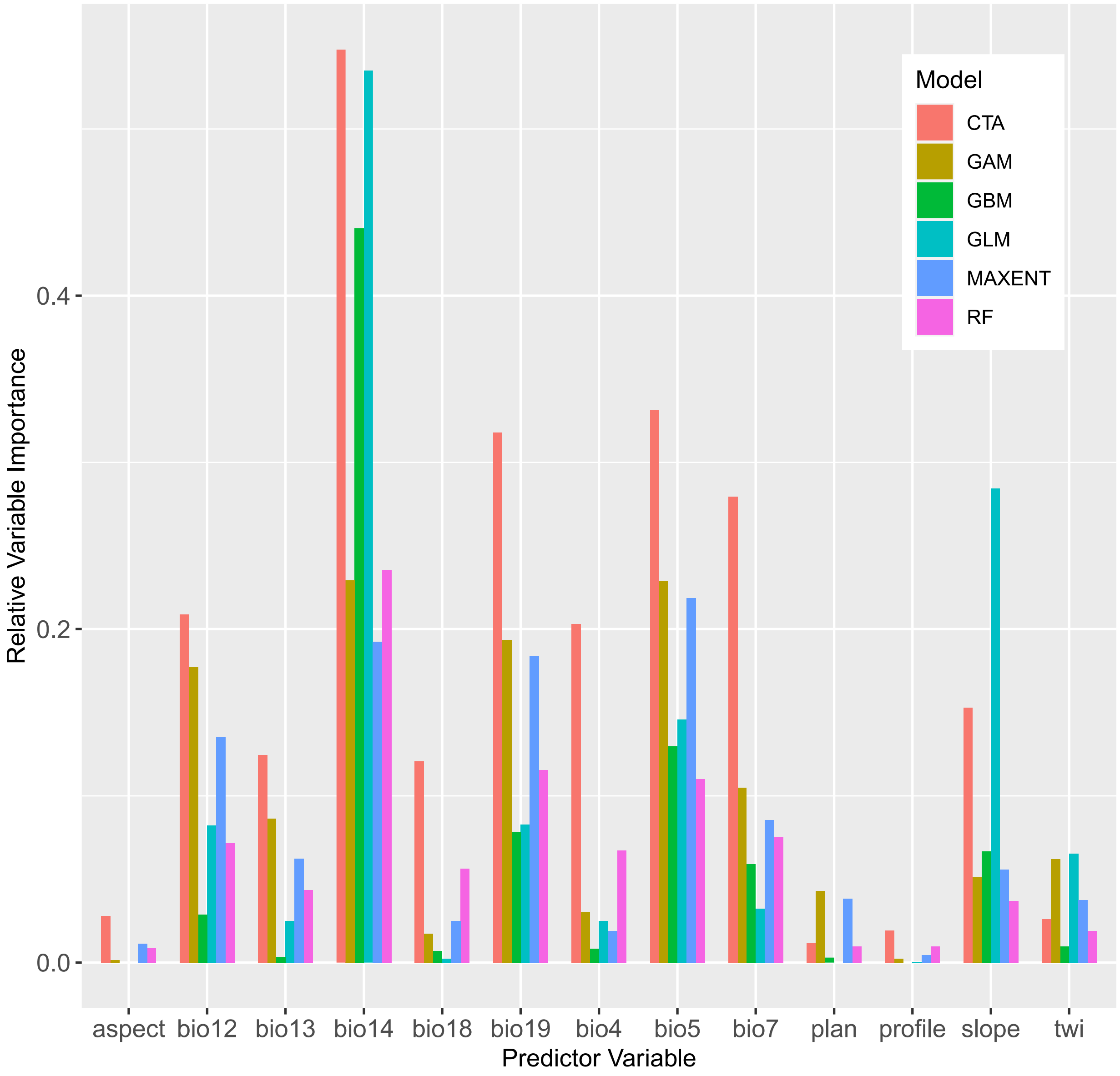

Supplement: S3 Appendix — (PDF) [file pone.0275360.s003.pdf]
