# Supplementary material for: Assessing distribution changes of selected native and alien invasive plant species under changing climatic conditions in Nyeri County, Kenya
Source: PLoS One. 2022 Oct 3;17(10):e0275360. doi: 10.1371/journal.pone.0275360 (PMC9529121; doi:10.1371/journal.pone.0275360)
Supplement: S4 Appendix — (PDF) [file pone.0275360.s004.pdf]

S4 Appendix. Forest outline layer overlaid on the current continuous and binary suitability maps.

After intersecting the forestland layer (including both fragmented forestland and the Aberdare and Mt. Kenya national reserves), also shown in Fig 1, with the species binary suitable areas in QGIS software, the portions of forested areas out of an approximate total area of 1,580 Km^2 that intersected the predicted species suitable areas were as follows: ~562 Km^2 (36%) for *C. decapetala*, ~123 Km^2 (~8%) for *L. camara*, ~206 Km^2 (13%) for *O. stricta*, ~573 Km^2 (36%) for *S. didymobotrya*, and ~292 Km^2 (18%) for *S. campylacanthum.* Notably, the Aberdare ecosystem to the west and the Mt. Kenya ecosystem to the east intersected the potential suitable areas around their edges. It is expected that the potential suitable area will likely continue to increase to large extents within these ecosystems under future climate scenarios.

Fig 1. Forest outline layer overlaid on the continuous and binary suitability maps for the current climate. On the binary maps, the red hue (1), denotes potential suitable areas while blue hue (0) denotes unsuitable areas. The overlaid forest layer is represented by the gray outline symbol and a white fill which shows on the maps as light blue and light red in areas where the layer intersects the unsuitable and suitable areas respectively. The black outline denotes the nyeri subcounty boundaries namely: *i, ii, iii, iv, v and vi which represents Kieni, Tetu, Othaya, Nyeri Town, Mathira and Mukurwe-ini sub-counties. The respective maps for the individual species are denoted as follows: (a) C. decapetala (Roth) Alston; (b) L. camara; (c) O. stricta; (d) S. didymobotrya; (e) S. campylacanthum Hochst. ex A. Rich.*

Data sources: Basemaps showing continuous and binary suitability maps were produced by the author; Administrative boundaries were obtained from (GADM database, www.gadm.org, license: https://gadm.org/license.html); Forest/tree cover layer was obtained from: SERVIR GLOBAL data catalog (https://servirglobal.net/Data-and-Maps) under CC BY 4.0 license.

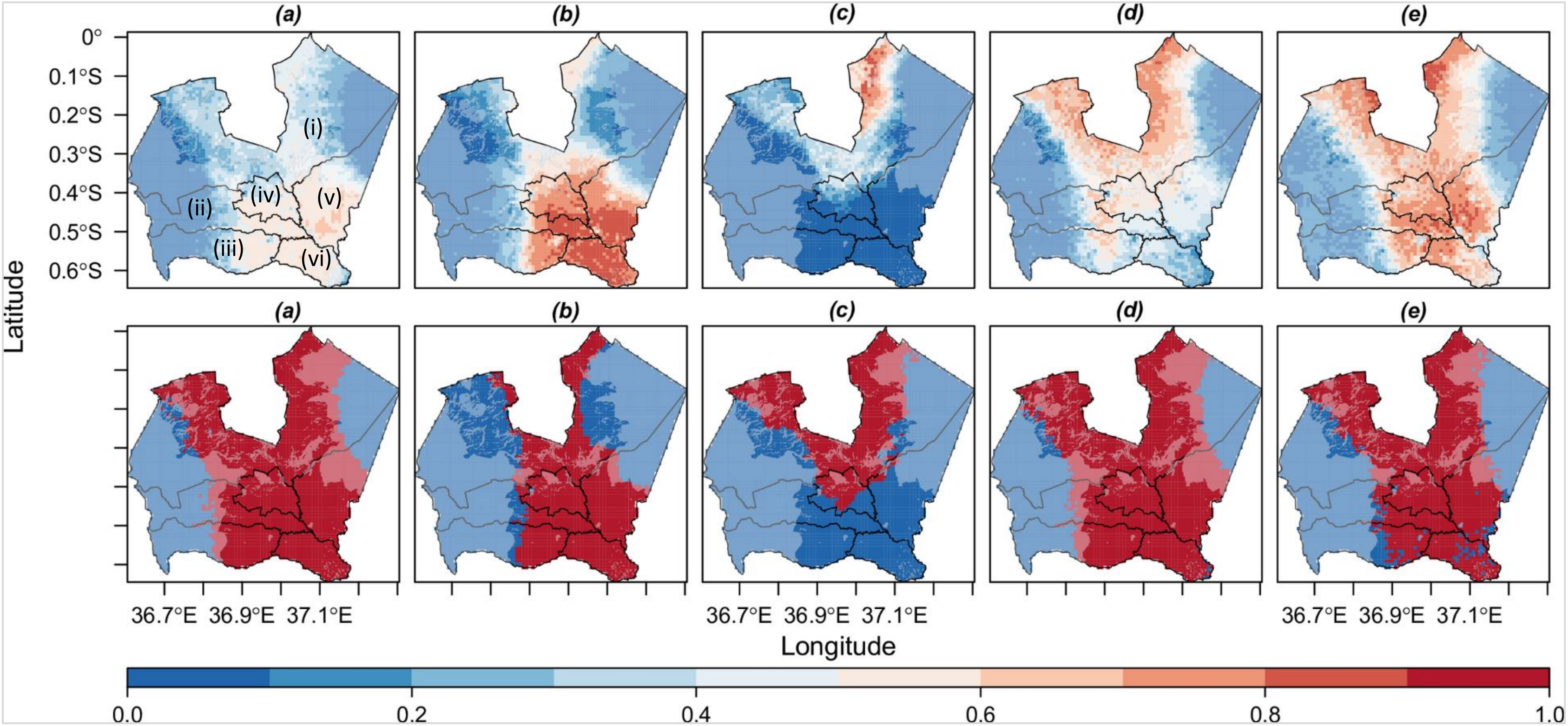

(a)
(b)
(c)
(d)
(e)
0°
0.1°S
0.2°S
0.3°S
0.4°S
0.5°S
0.6°S
(i)
(ii)
(iii)
(iv)
(v)
(vi)
Latitude
36.7°E
36.9°E
37.1°E
Longitude
0.0
0.2
0.4
0.6
0.8
1.0
