# Supplementary material for: Assessing distribution changes of selected native and alien invasive plant species under changing climatic conditions in Nyeri County, Kenya
Source: PLoS One. 2022 Oct 3;17(10):e0275360. doi: 10.1371/journal.pone.0275360 (PMC9529121; doi:10.1371/journal.pone.0275360)

S5 Appendix. Species future ensemble predictions and clamping masks for the periods 2050s and 2070s.

Fig1. Species future ensemble prediction binary median maps and respective clamping masks for the future periods 2050s and 2070s. suitable areas on binary maps are shown in red (1.0) while unsuitable areas are shown in blue (0.0). Dark green color value (1.0) indicate areas where models predictions are highly uncertain while towards the bottom value (0.0) light green to light shade of pink shows low uncertainty. Individual GCM data are denoted as follows: bcc, BCC-CSM1.1(m); esm2g, GFDL-ESM2G; hadgem2, Hadgem2-ES; ipsl, IPSL-CM5A-MR; miroc, MIROC-ESM-CHEM; and ncar, NCAR-CCSM4.

Data sources: Basemaps showing binary suitability maps and prediction clamping masks were produced by the author. Administrative boundary layer: obtained from GADM database (www.gadm.org) under CC BY 4.0 license (https://gadm.org/license.html);

(a) ***C. decapetala (Roth) Alston***

Ensemble future predictions binary maps for the period 2050s

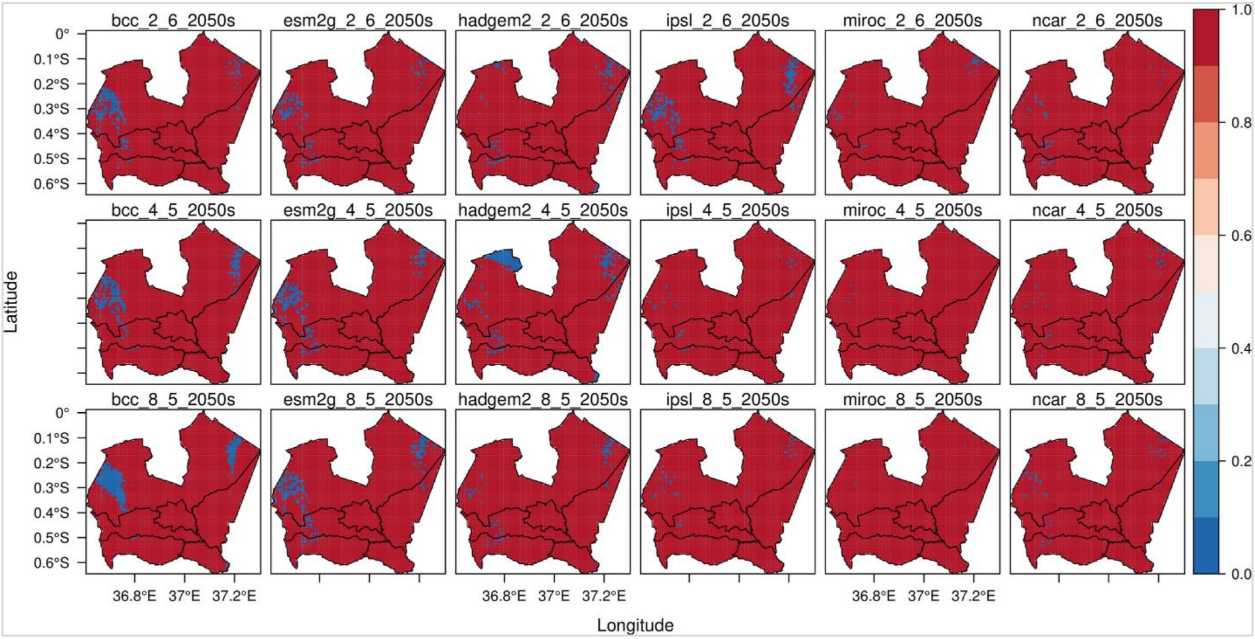

Ensemble model prediction clamping masks for the period 2050s

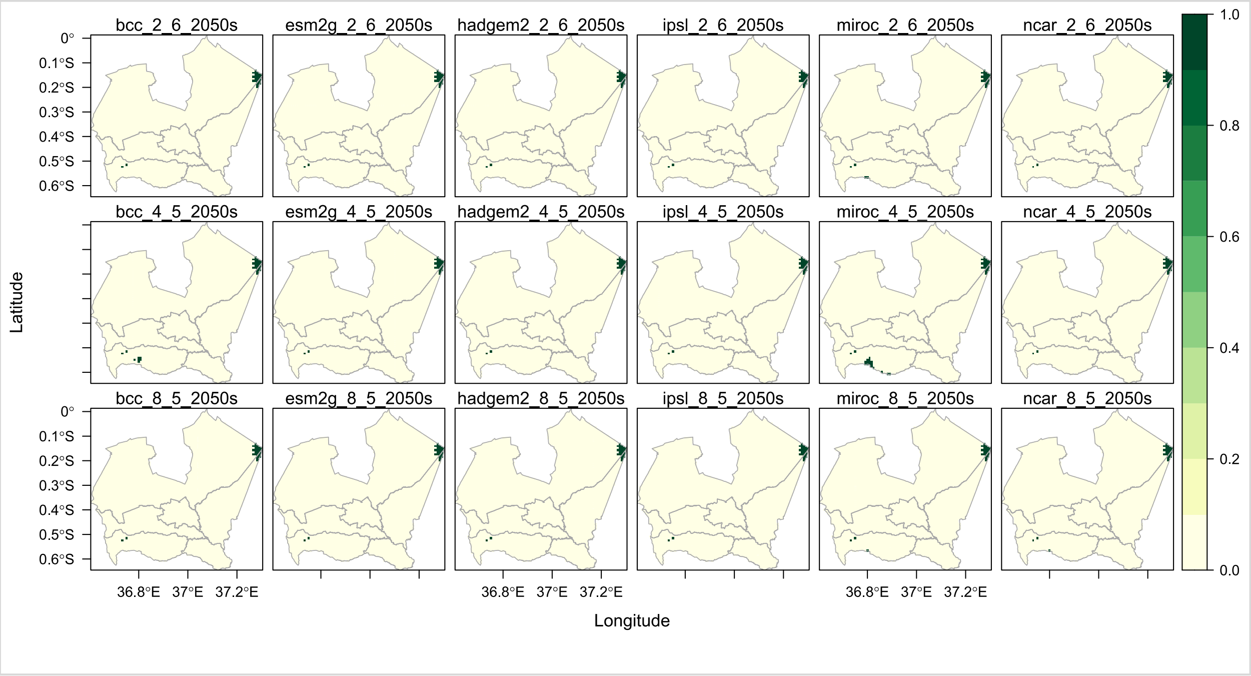

C. decapetala (Roth) Alston

Ensemble future predictions binary maps for the period 2070s

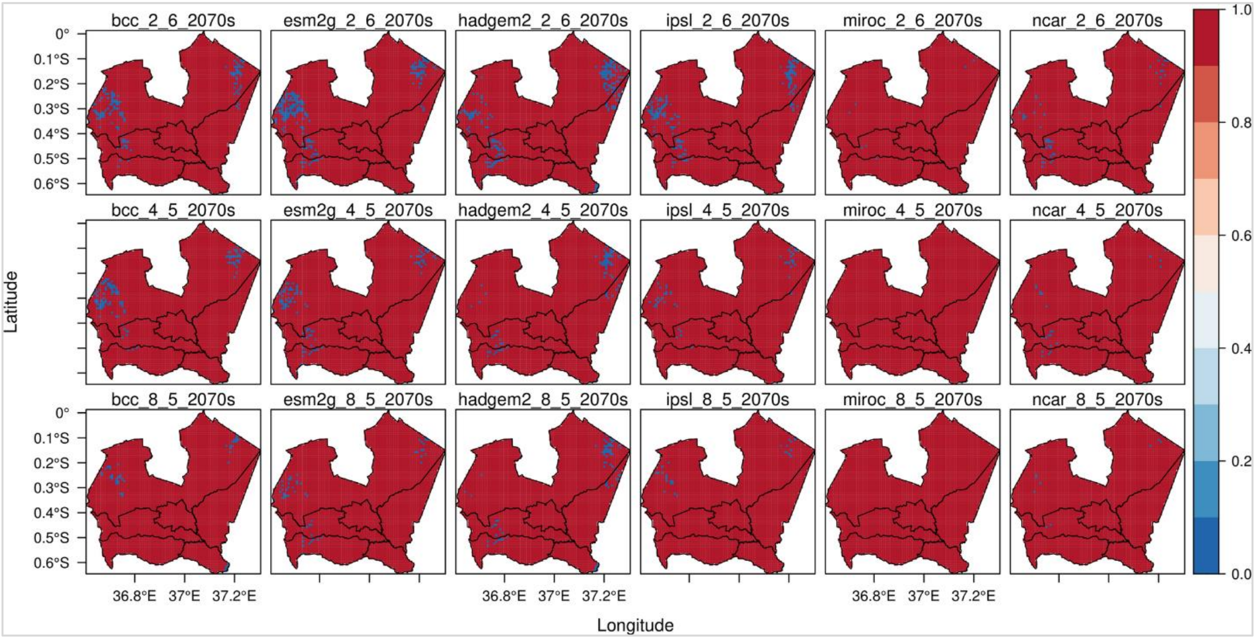

Ensemble model prediction clamping masks for the period 2070s

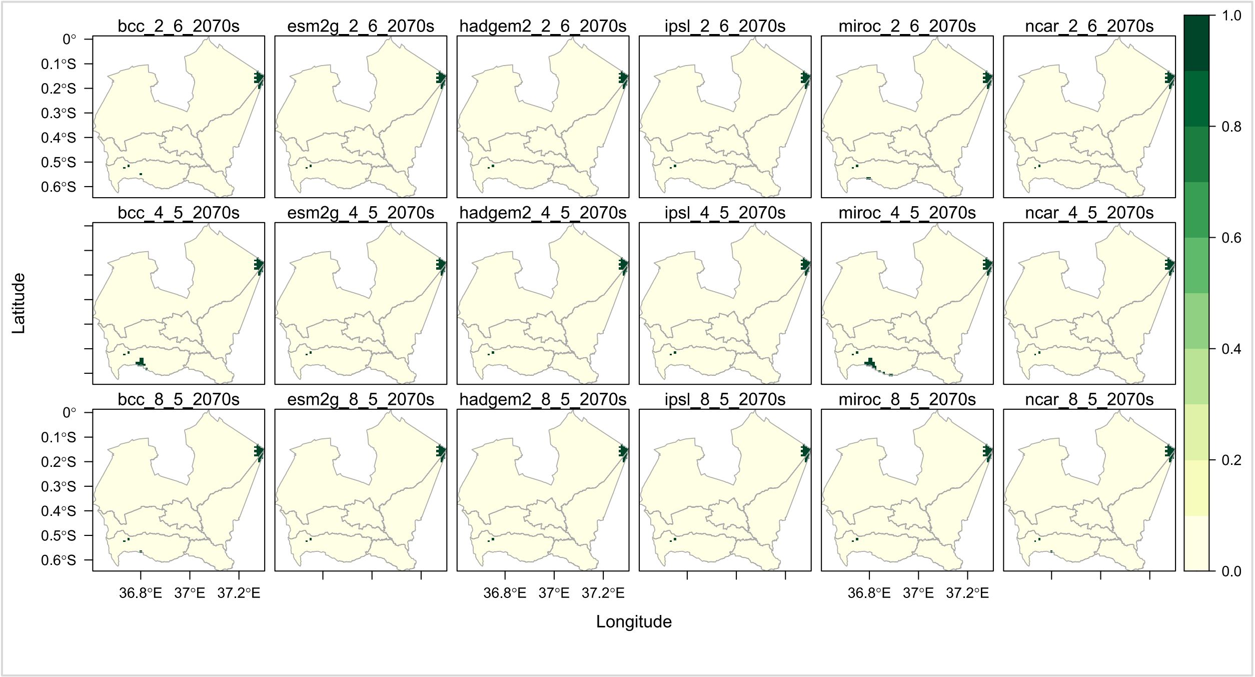

(b) *L. camara;*

Ensemble future predictions binary maps for the period 2050s

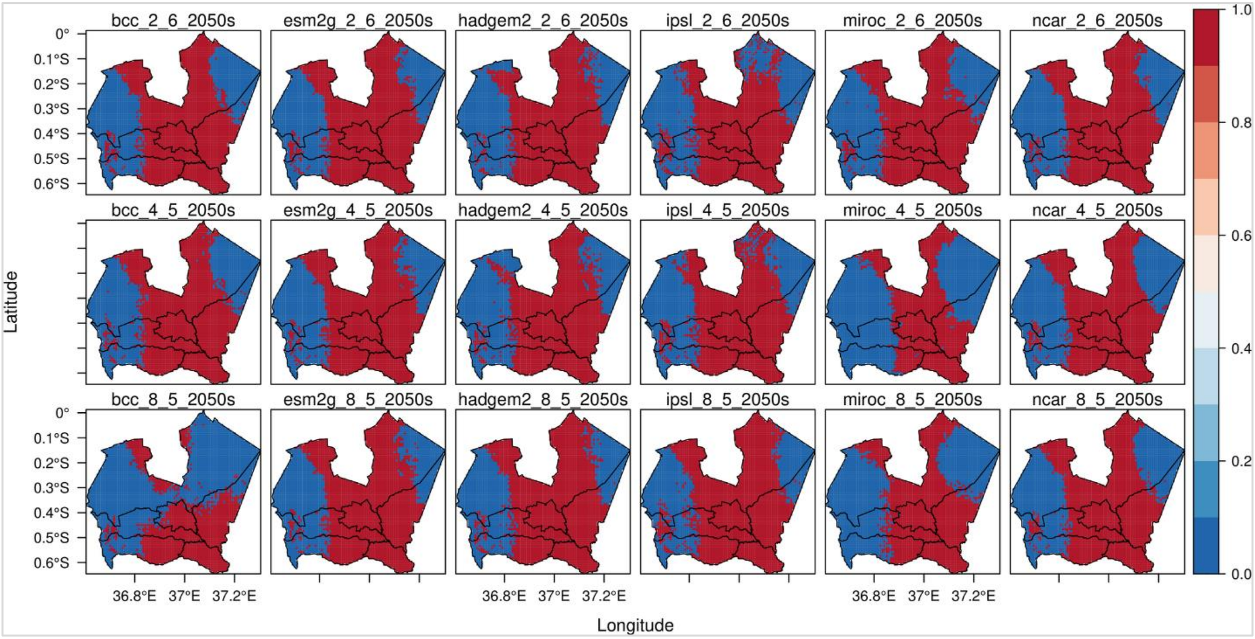

Ensemble model prediction clamping masks for the period 2050s

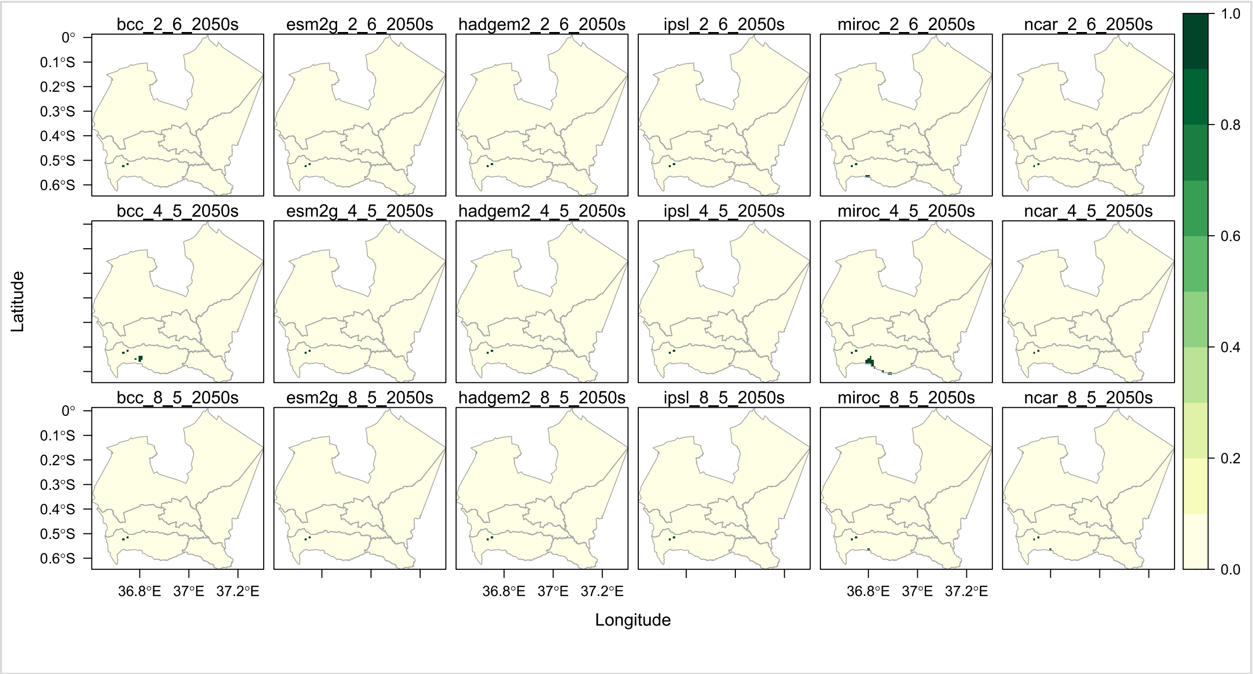

L. camara;

Ensemble future predictions binary maps for the period 2070s

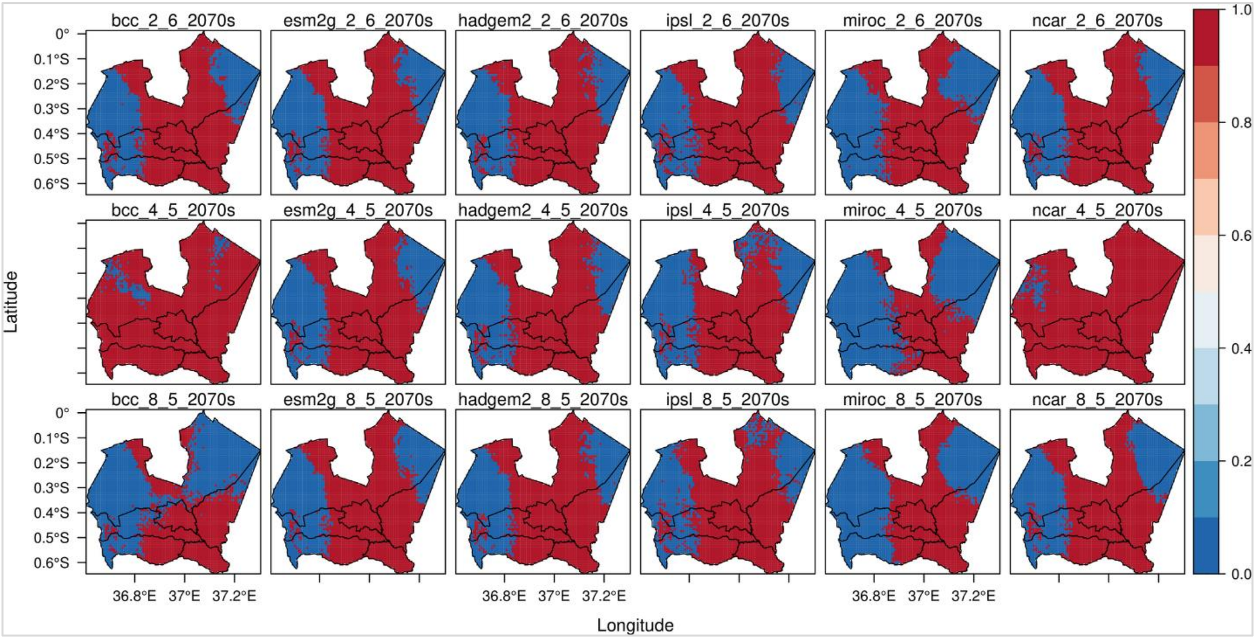

Ensemble model prediction clamping masks for the period 2070s

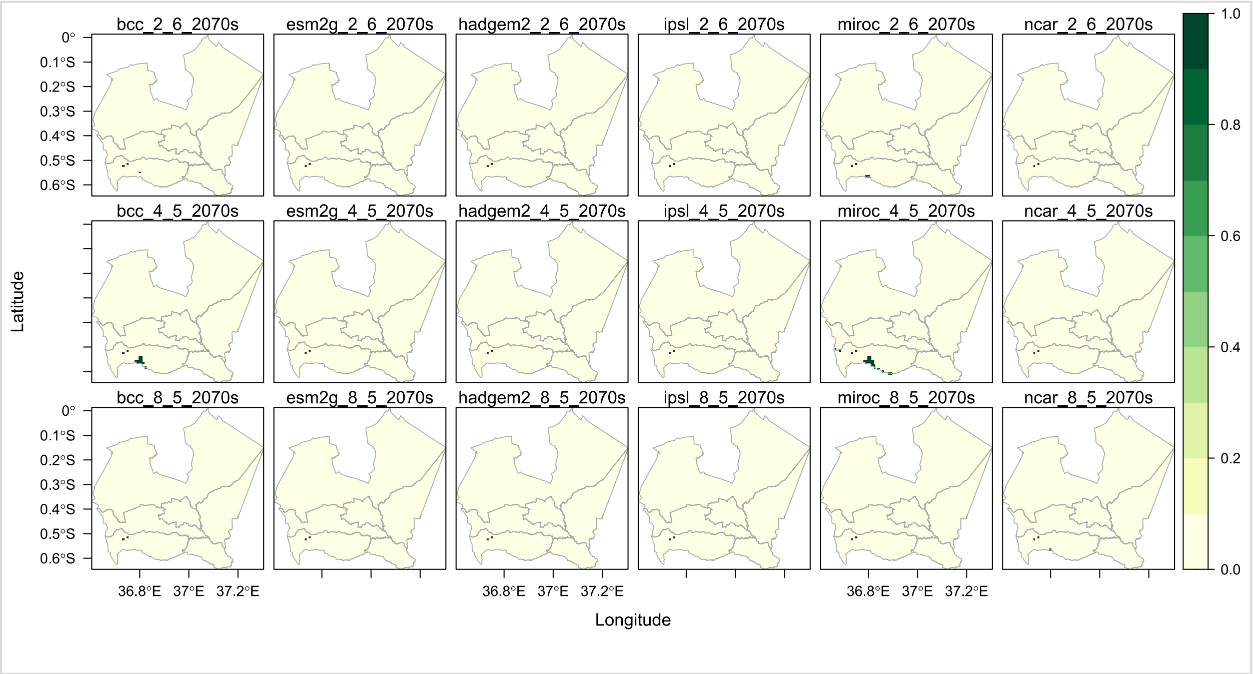

(c) *O. stricta;*

Ensemble future predictions binary maps for the period 2050s

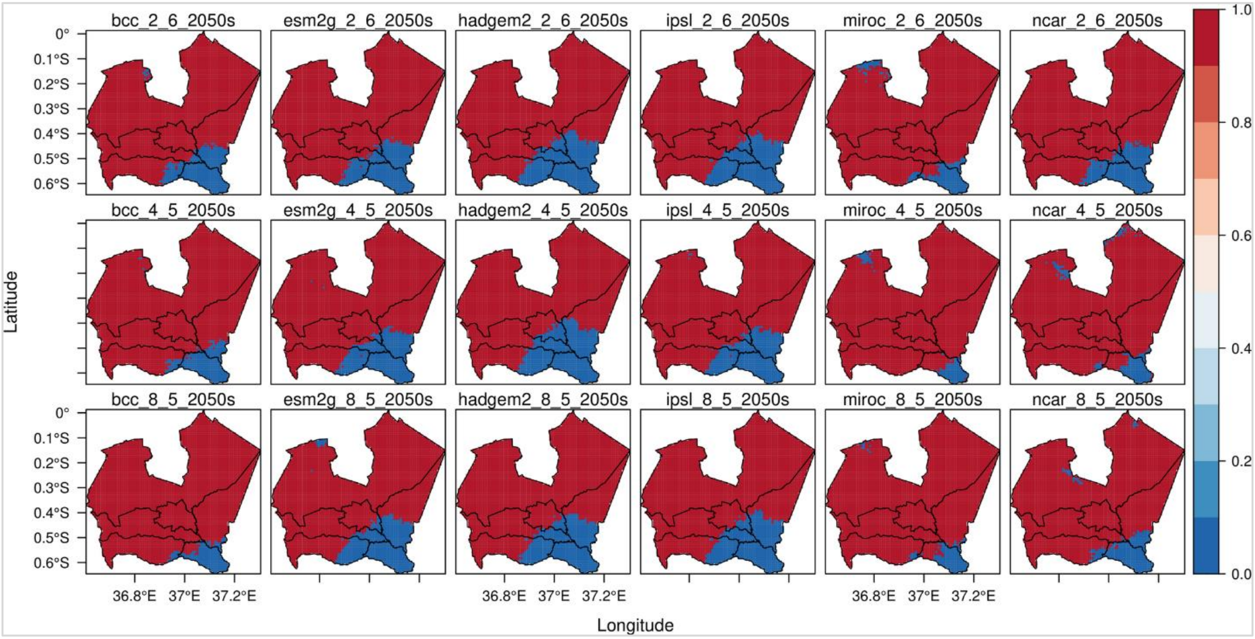

Ensemble model prediction clamping masks for the period 2050s

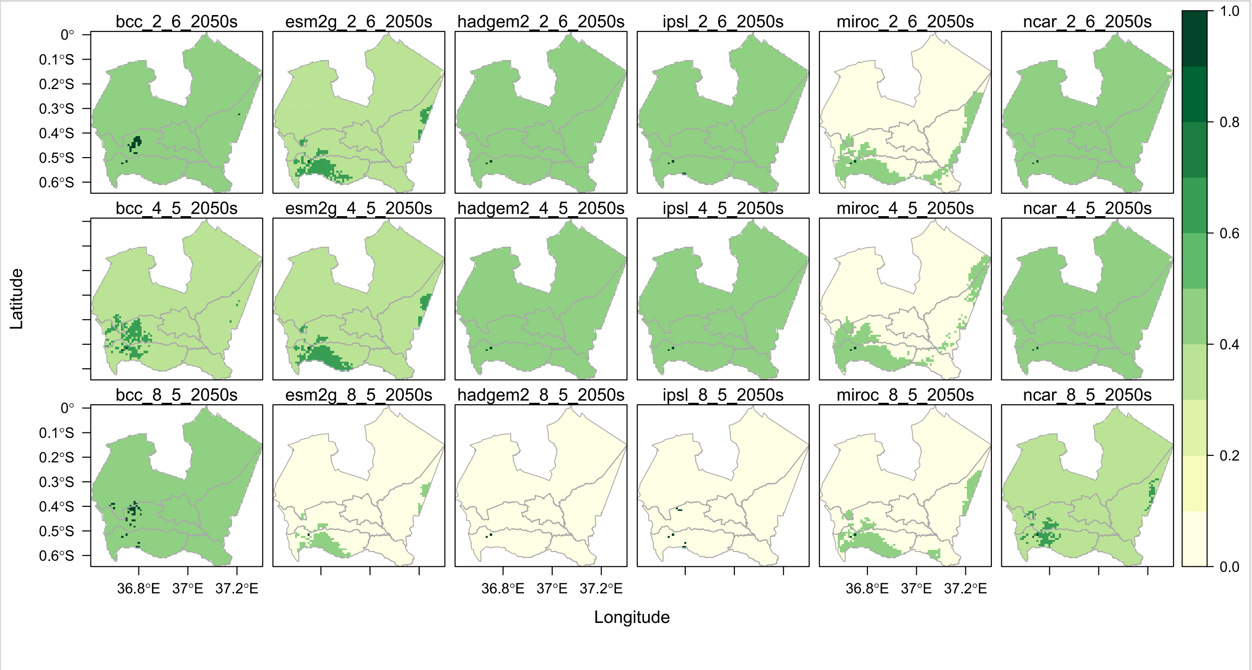

O. stricta;

Ensemble future predictions binary maps for the period 2070s

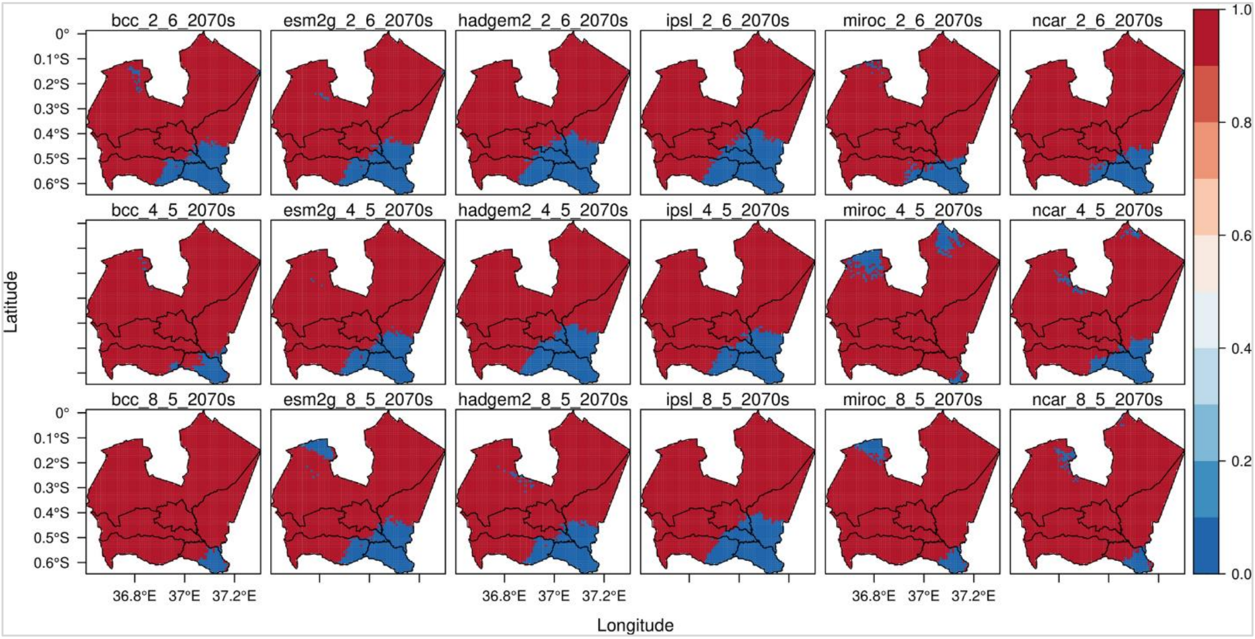

Ensemble model prediction clamping masks for the period 2070s

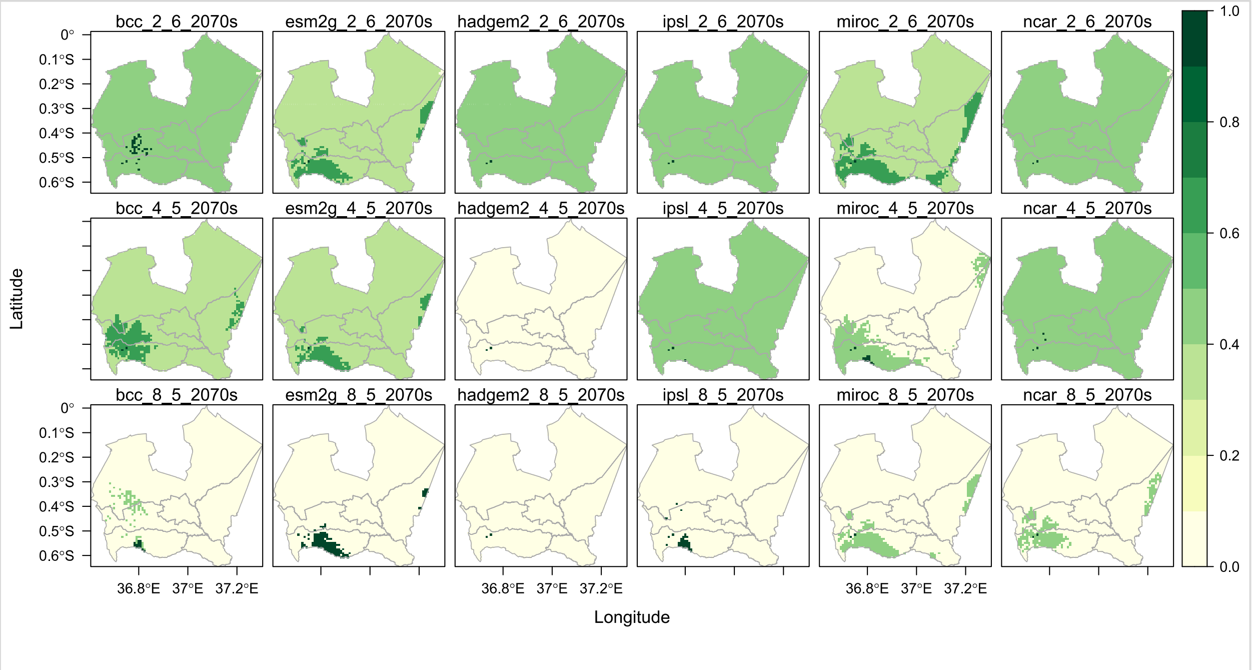

(d) *S. didymobotrya;*

Ensemble future predictions binary maps for the period 2050s

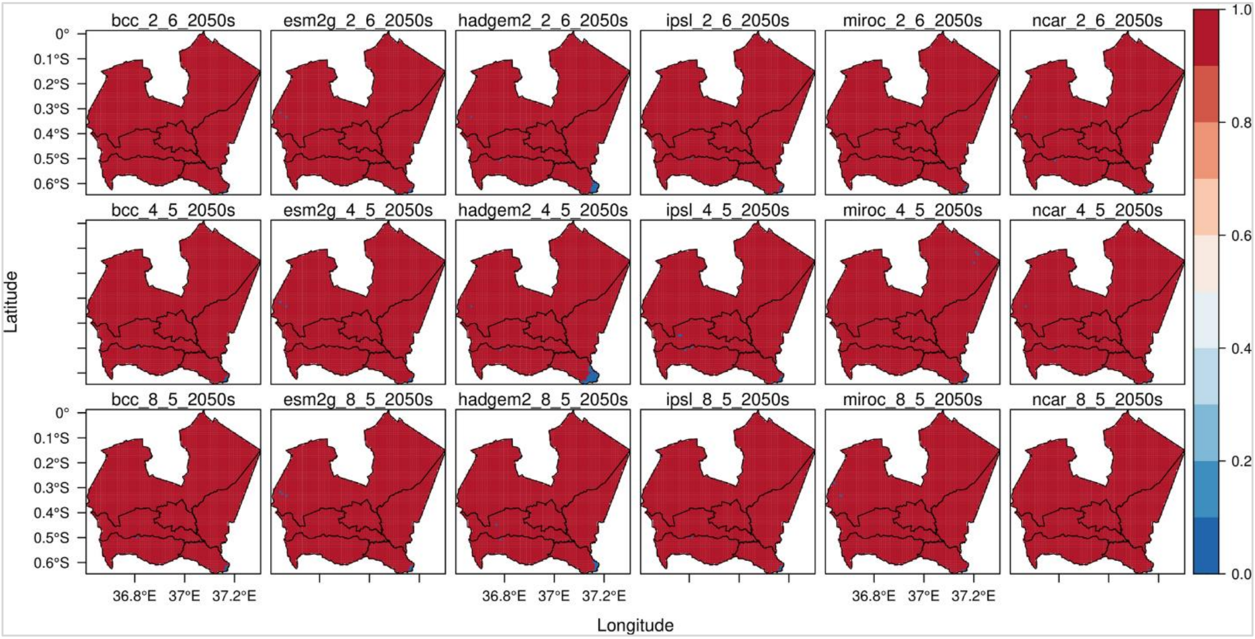

Ensemble model prediction clamping masks for the period 2050s

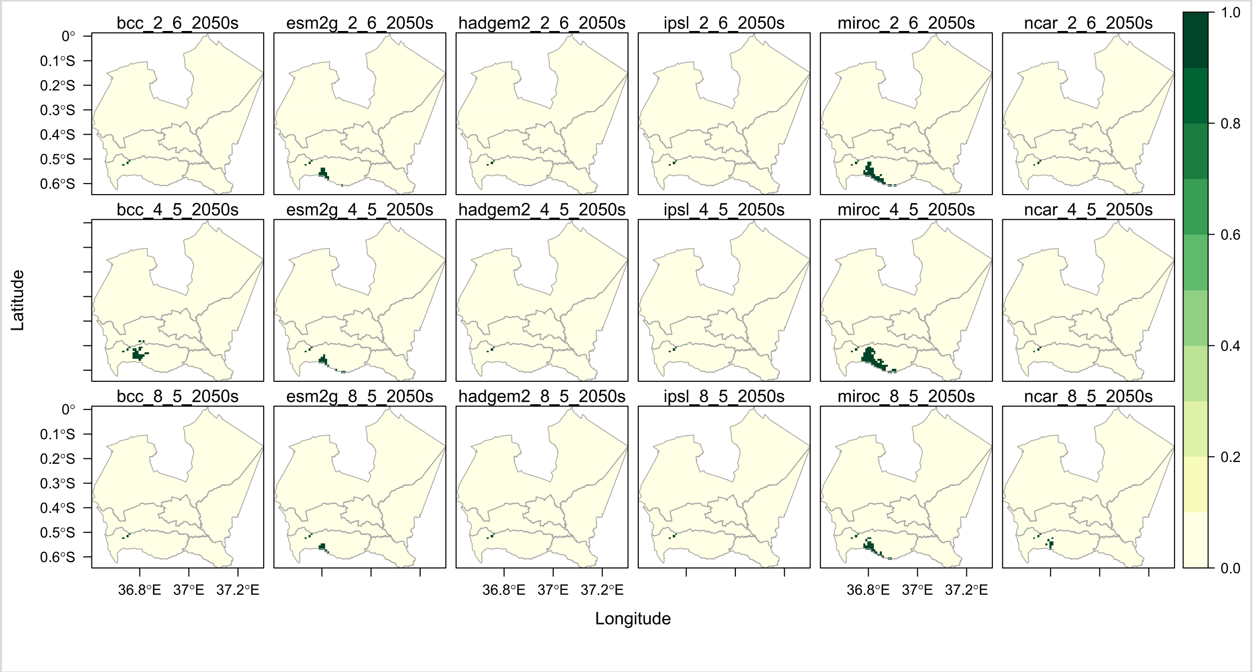

S. didymobotrya;

Ensemble future predictions binary maps for the period 2070s

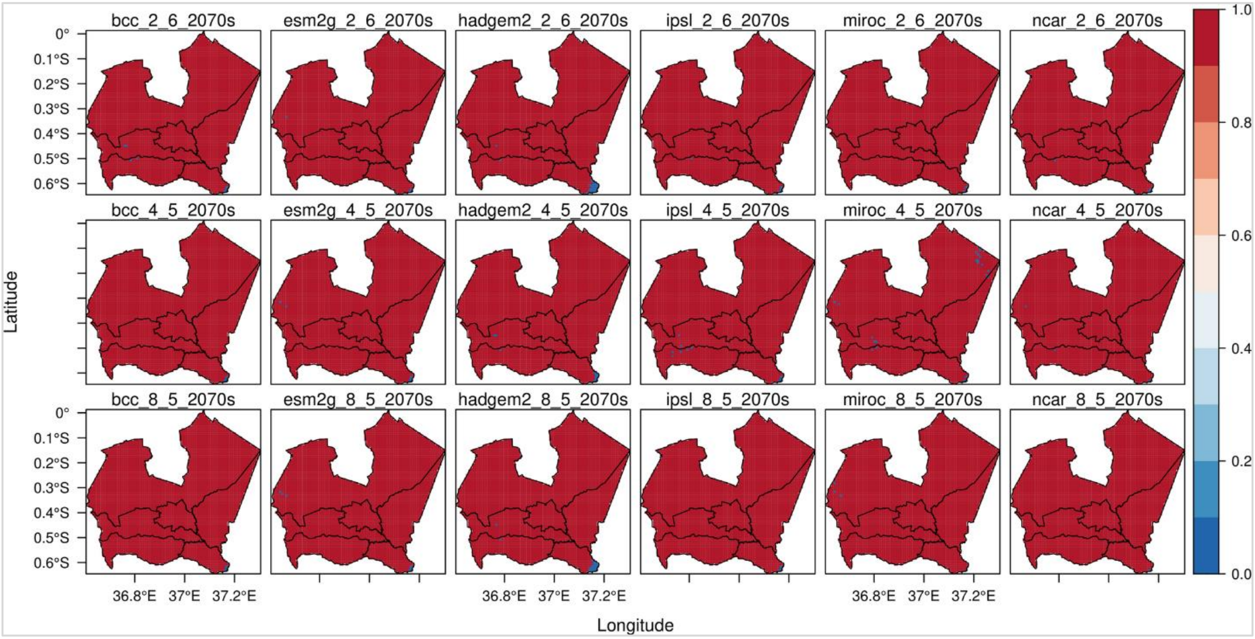

Ensemble model prediction clamping masks for the period 2070s

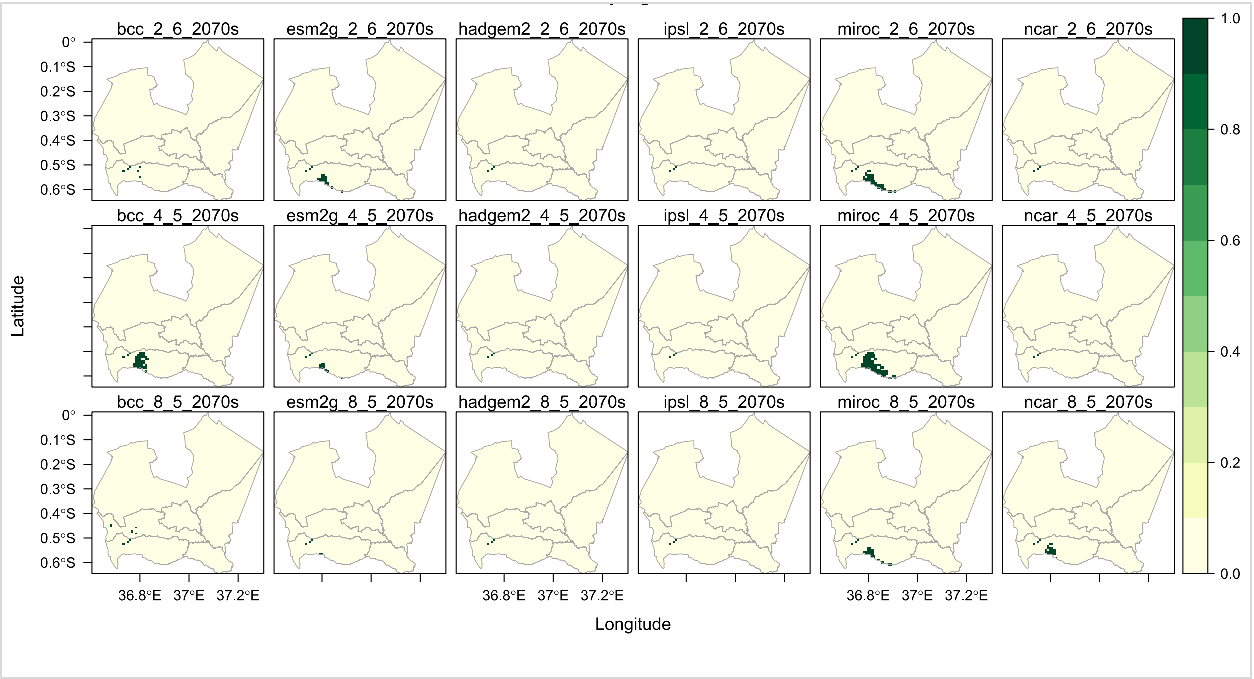

(e) *S. campylacanthum Hochst. ex A. Rich.*

Ensemble future predictions binary maps for the period 2050s

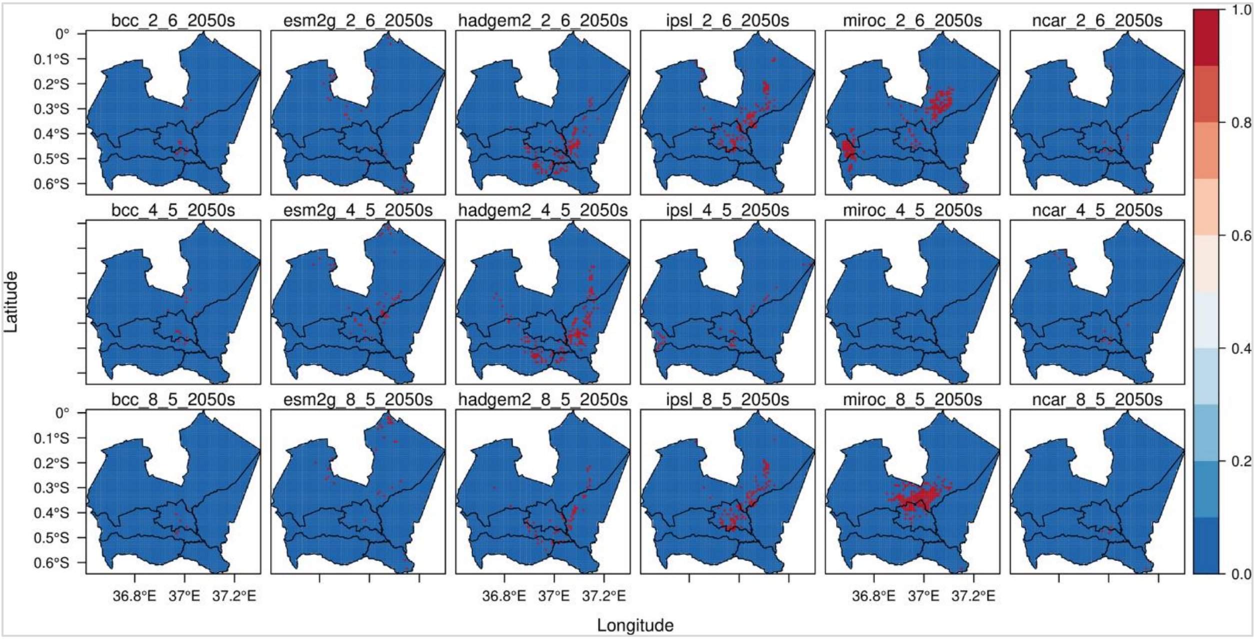

Ensemble model prediction clamping masks for the period 2050s

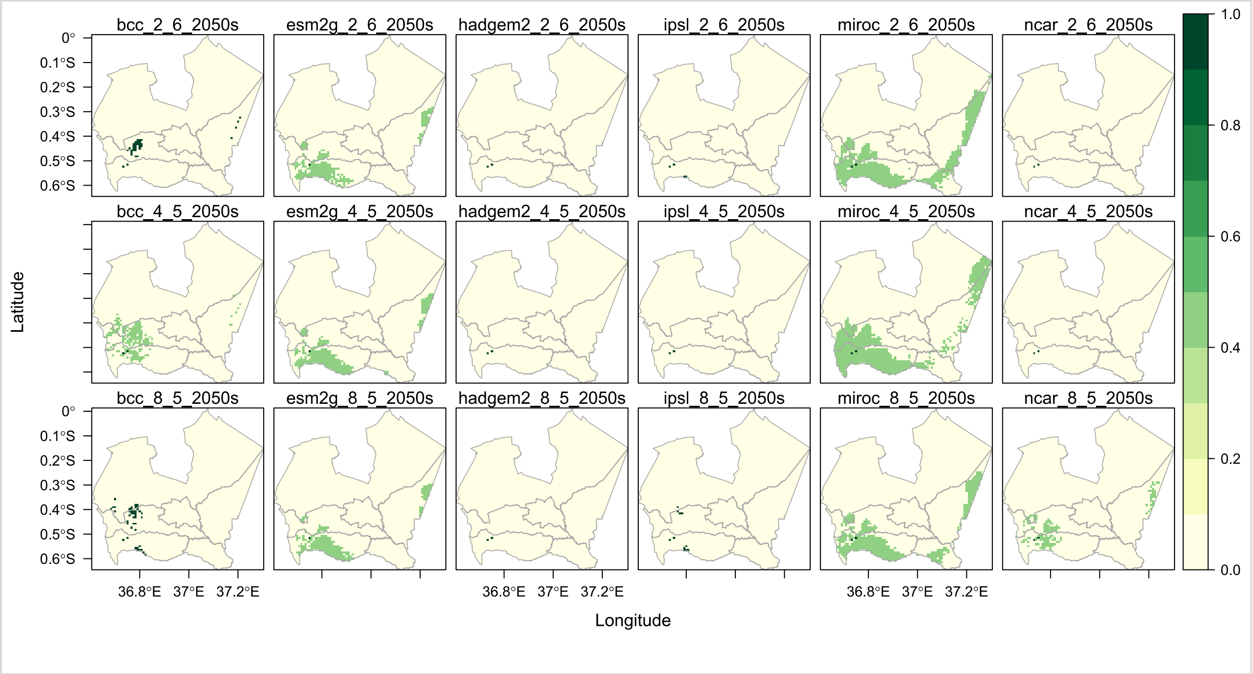

***S. campylacanthum* Hochst. ex A. Rich.**

Ensemble future predictions binary maps for the period 2070s

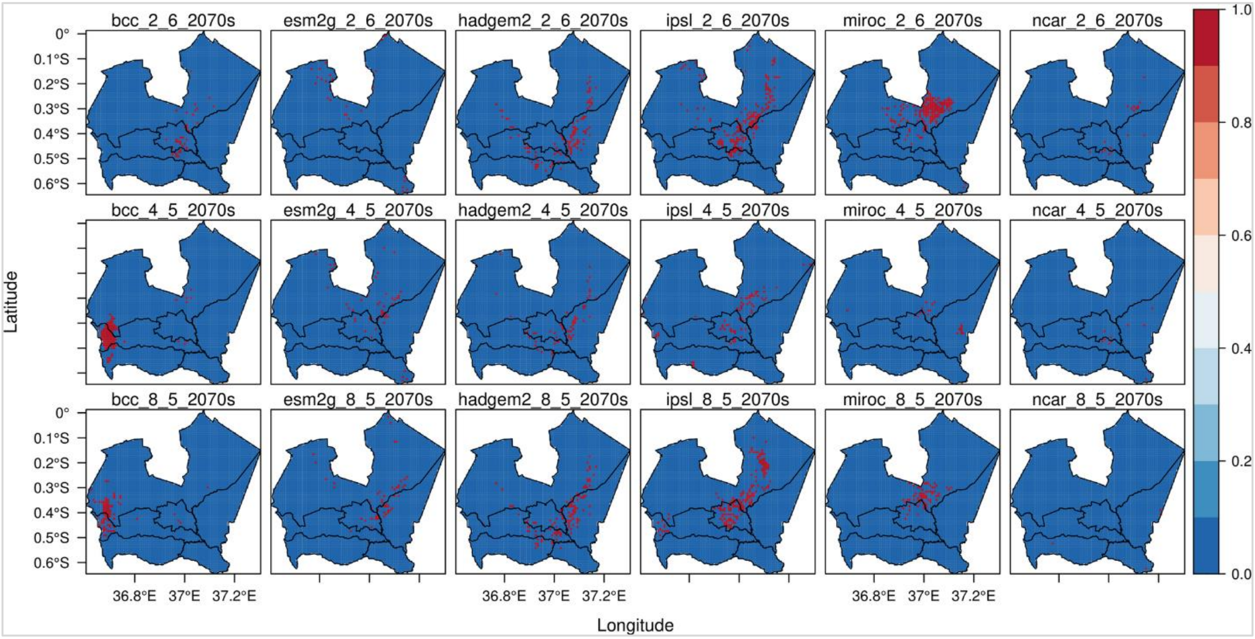

Ensemble model prediction clamping masks for the period 2070s

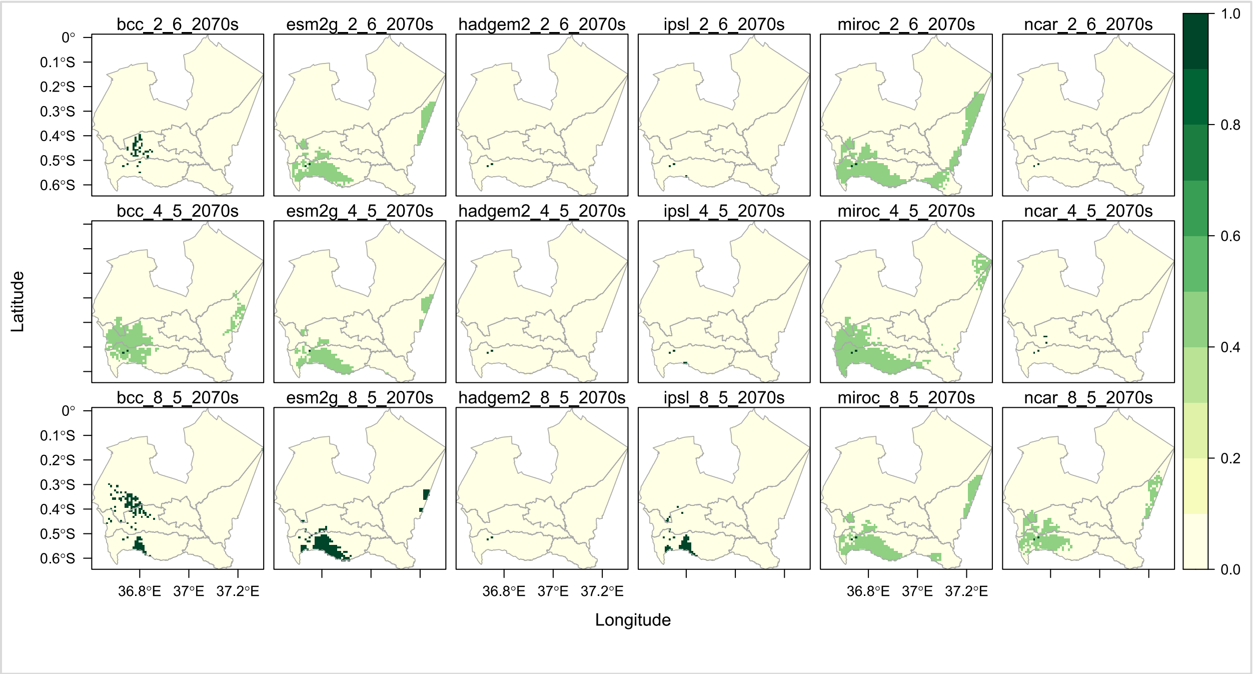

Supplement: S5 Appendix — (PDF) [file pone.0275360.s005.pdf]
