# Supplementary material for: Assessing distribution changes of selected native and alien invasive plant species under changing climatic conditions in Nyeri County, Kenya
Source: PLoS One. 2022 Oct 3;17(10):e0275360. doi: 10.1371/journal.pone.0275360 (PMC9529121; doi:10.1371/journal.pone.0275360)

S6 Appendix. Potential species habitat changes for the future periods 2050s and 2070s.

Fig 1. Habitat suitability changes based on individual GCM data under RCPs 2.6, 4.5 and 8.5 for 2050s and 2070s. -2 represents habitat loss, -1 represents suitable and stable in future, 0 represents not suitable, 1 represents habitat gain. The GCM models are denoted as follows: *bcc*, BCC-CSM1.1(m); *esm2g*, GFDL-ESM2G; *hadgem2*, Hadgem2-ES; *ipsl*, IPSL-CM5A-MR; *miroc*, MIROC-ESM-CHEM; and *ncar*, NCAR-CCSM4. *Data Source: Basemap showing habitat suitability changes were produced by the Author. Administrative Boundary Layer: obtained from GADM database (www.gadm.org) under CC BY 4.0 license (https://gadm.org/license.html);*

a) ***C. decapetala*** **(Roth) Alston**

Potential range size changes - 2050

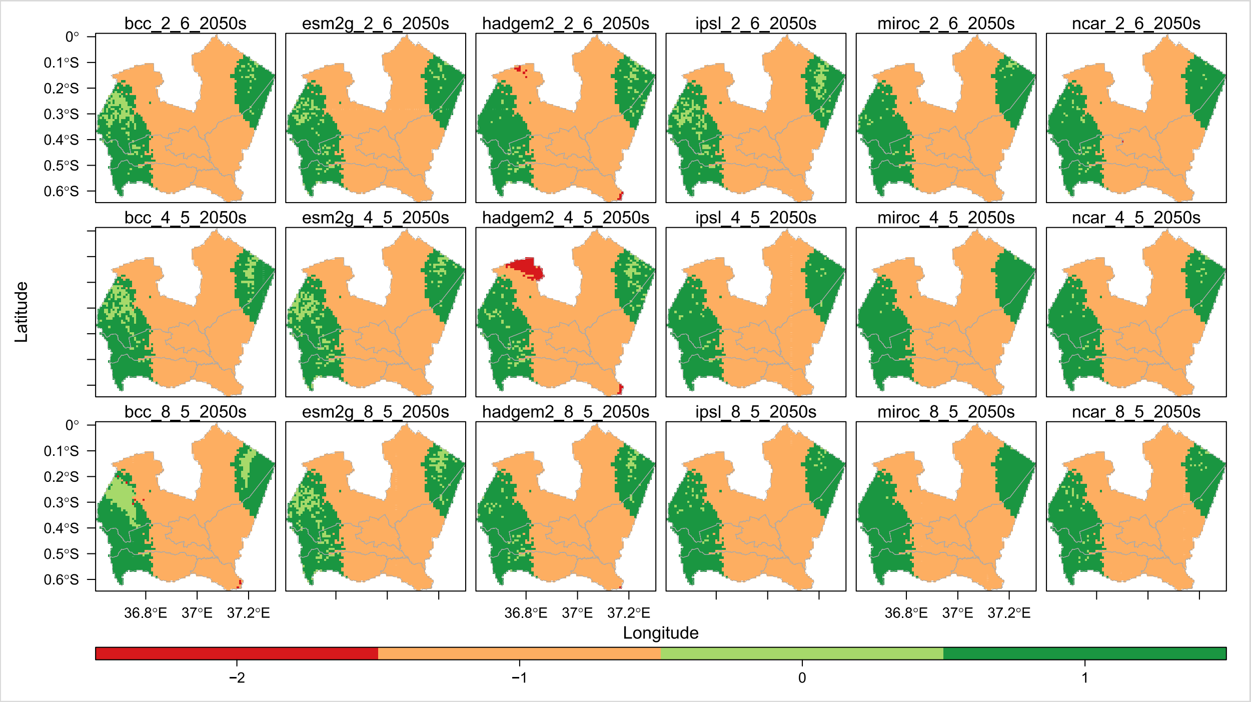

Potential range size changes - 2070

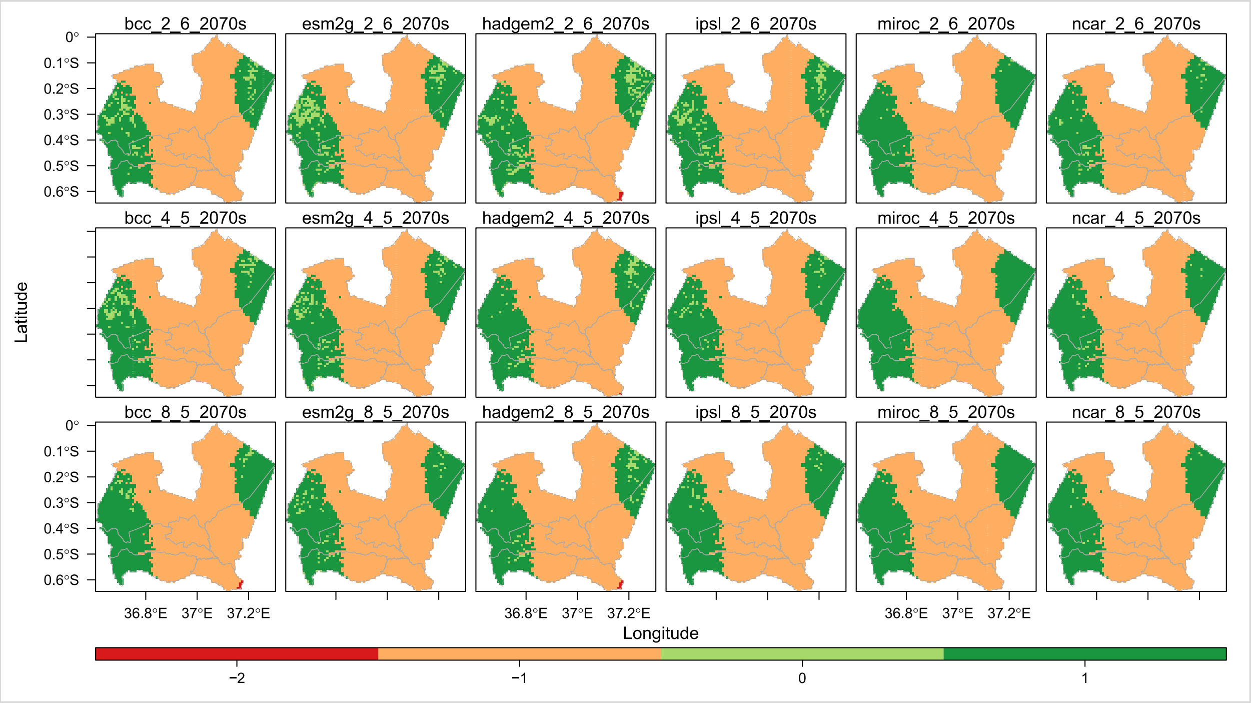
bcc_2_6_2070s
esm2g_2_6_2070s
hadgem2_2_6_2070s
ipsl_2_6_2070s
miroc_2_6_2070s
ncar_2_6_2070s
bcc_4_5_2070s
esm2g_4_5_2070s
hadgem2_4_5_2070s
ipsl_4_5_2070s
miroc_4_5_2070s
ncar_4_5_2070s
bcc_8_5_2070s
esm2g_8_5_2070s
hadgem2_8_5_2070s
ipsl_8_5_2070s
miroc_8_5_2070s
ncar_8_5_2070s
0°
0.1°S
0.2°S
0.3°S
0.4°S
0.5°S
0.6°S
Latitude
36.8°E
37°E
37.2°E
Longitude
−2
−1
0
1

b) *L. camara;*

Potential range size changes - 2050

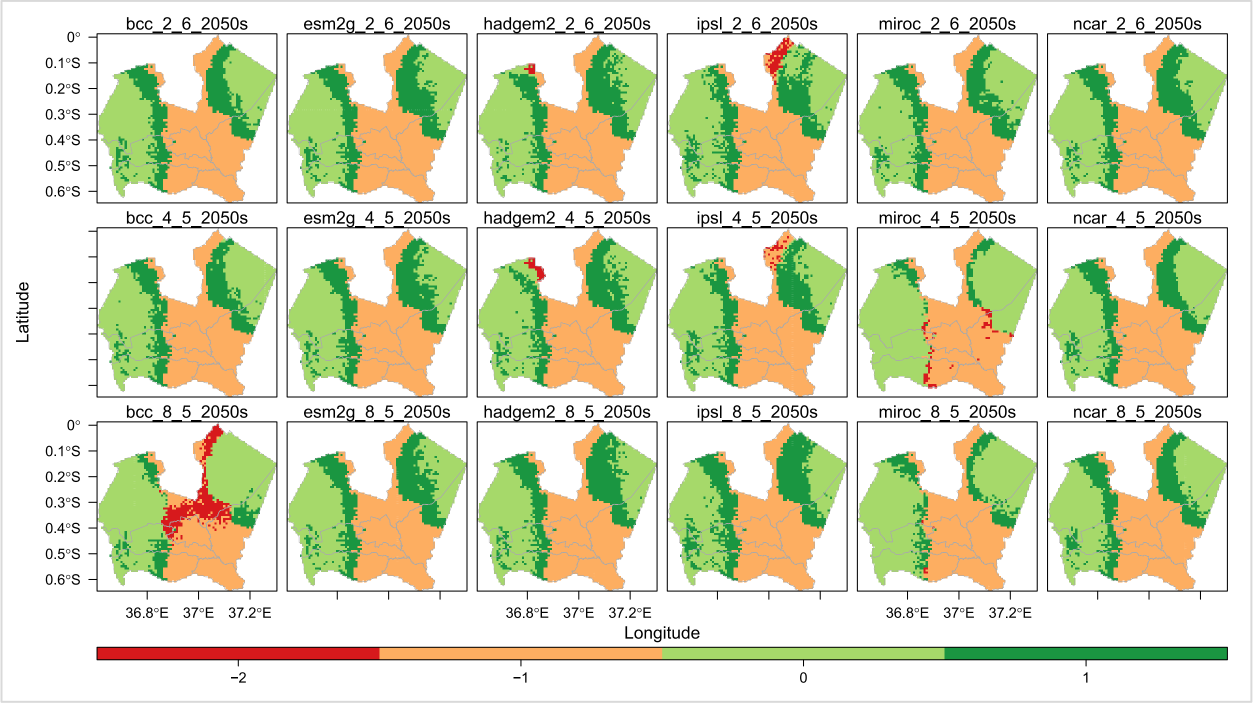

Potential range size changes - 2070

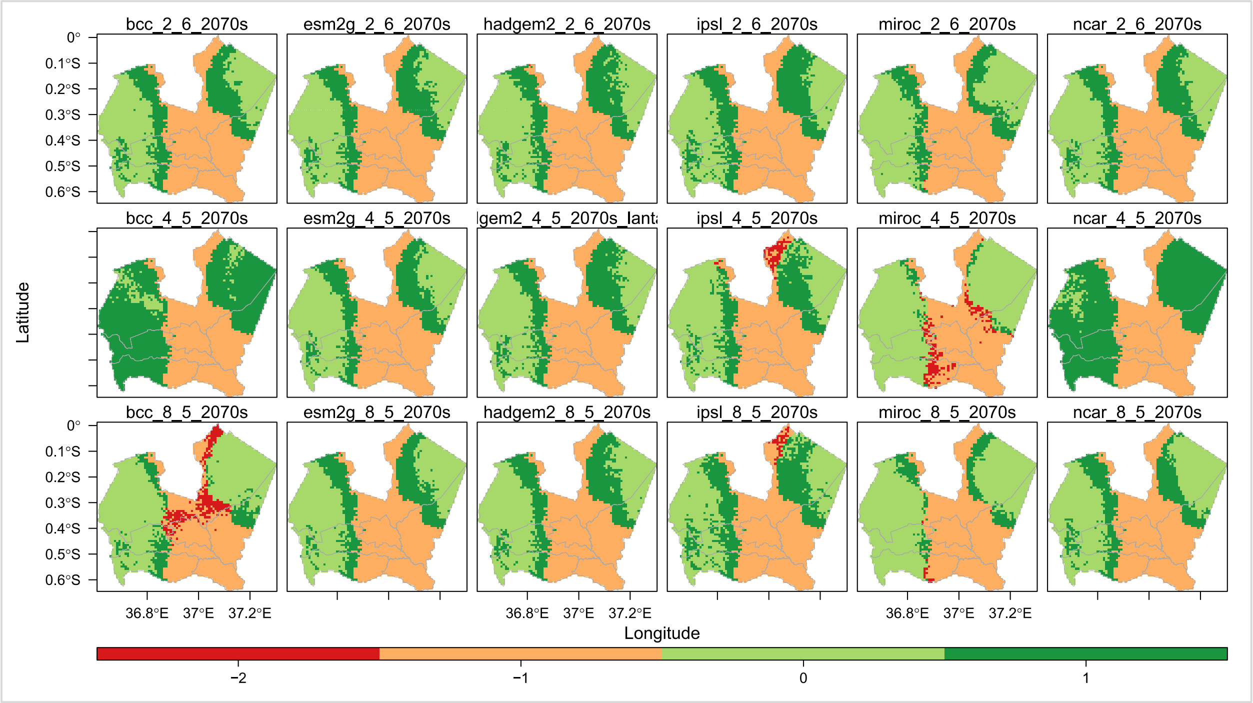

c) *O. stricta;*

Potential range size changes - 2050

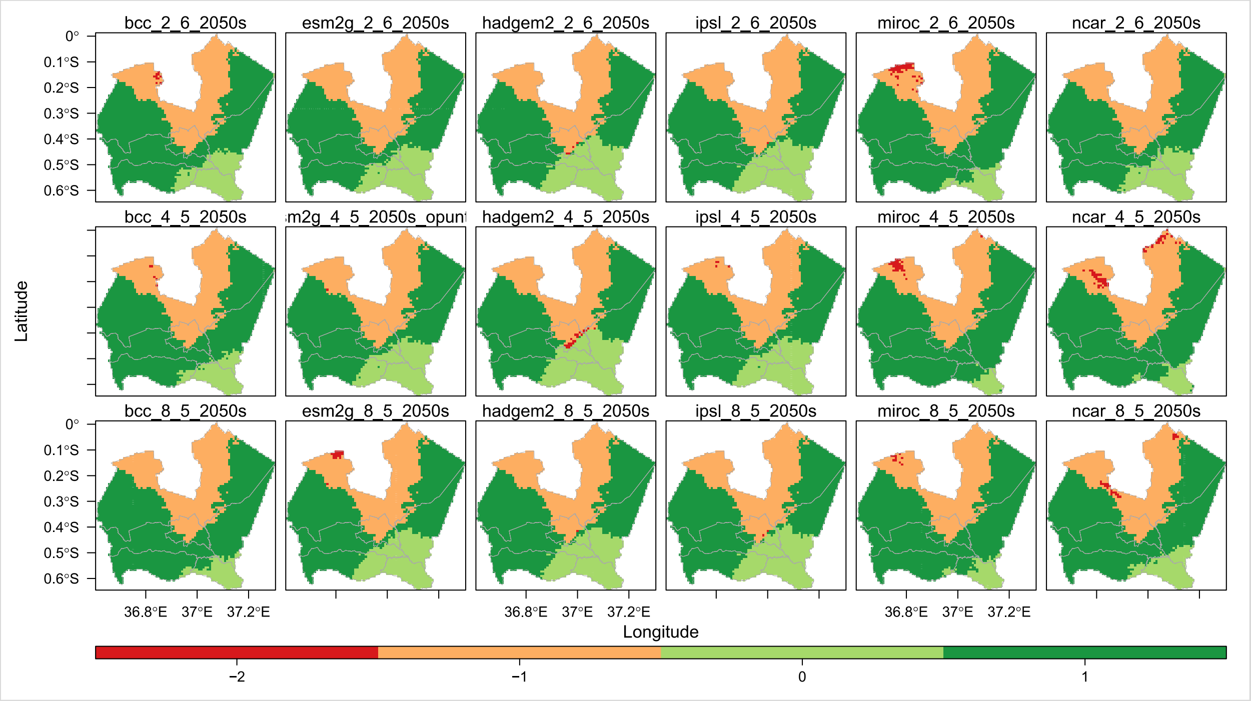

Potential range size changes - 2070

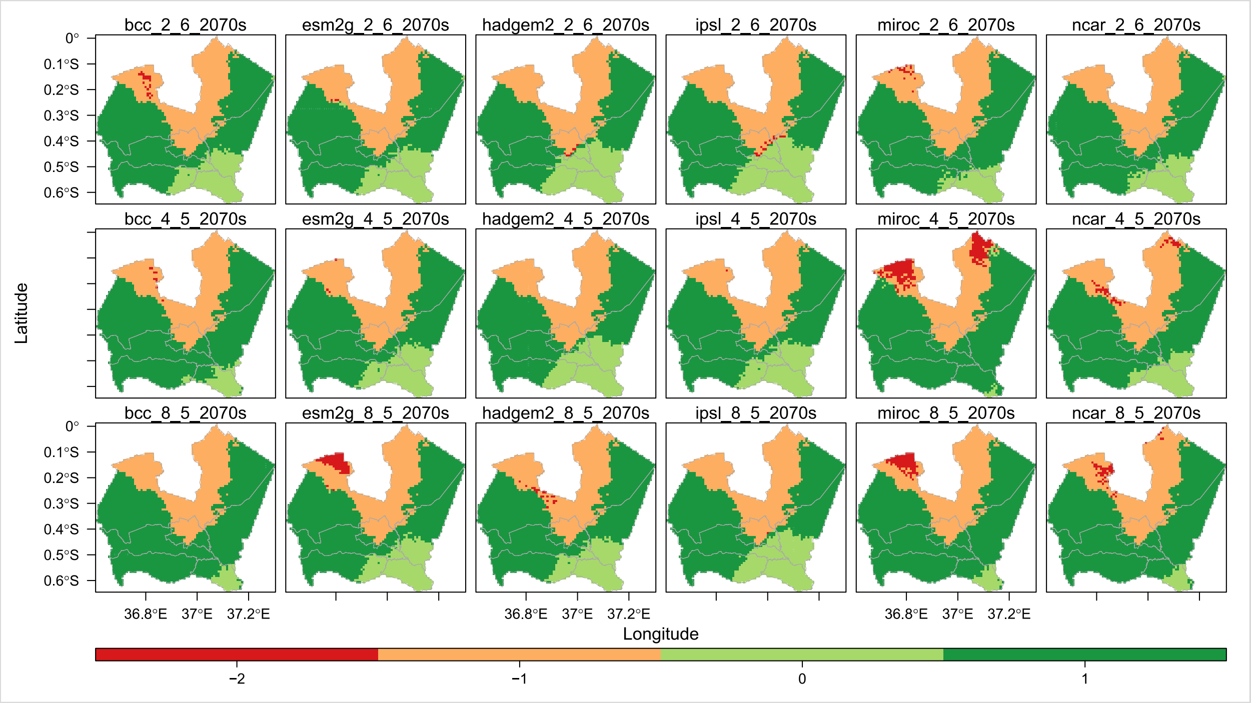

d) *S. didymobotrya;*

Potential range size changes - 2050

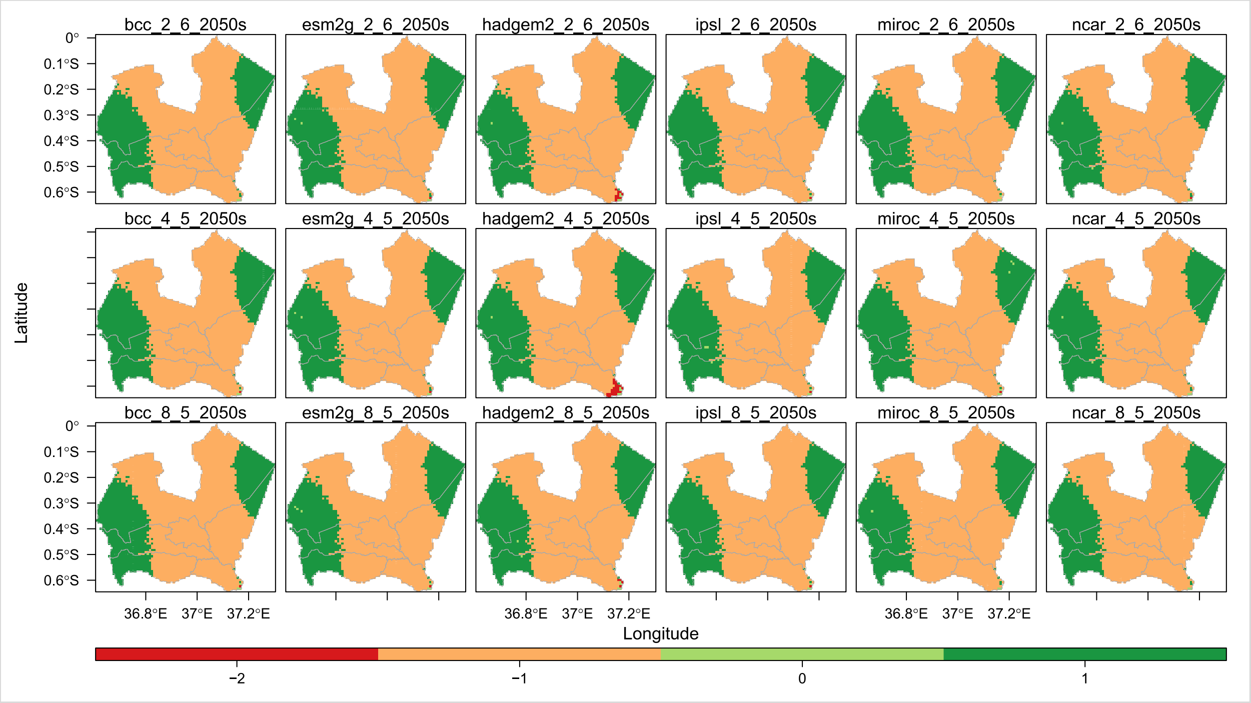

Potential range size changes - 2070

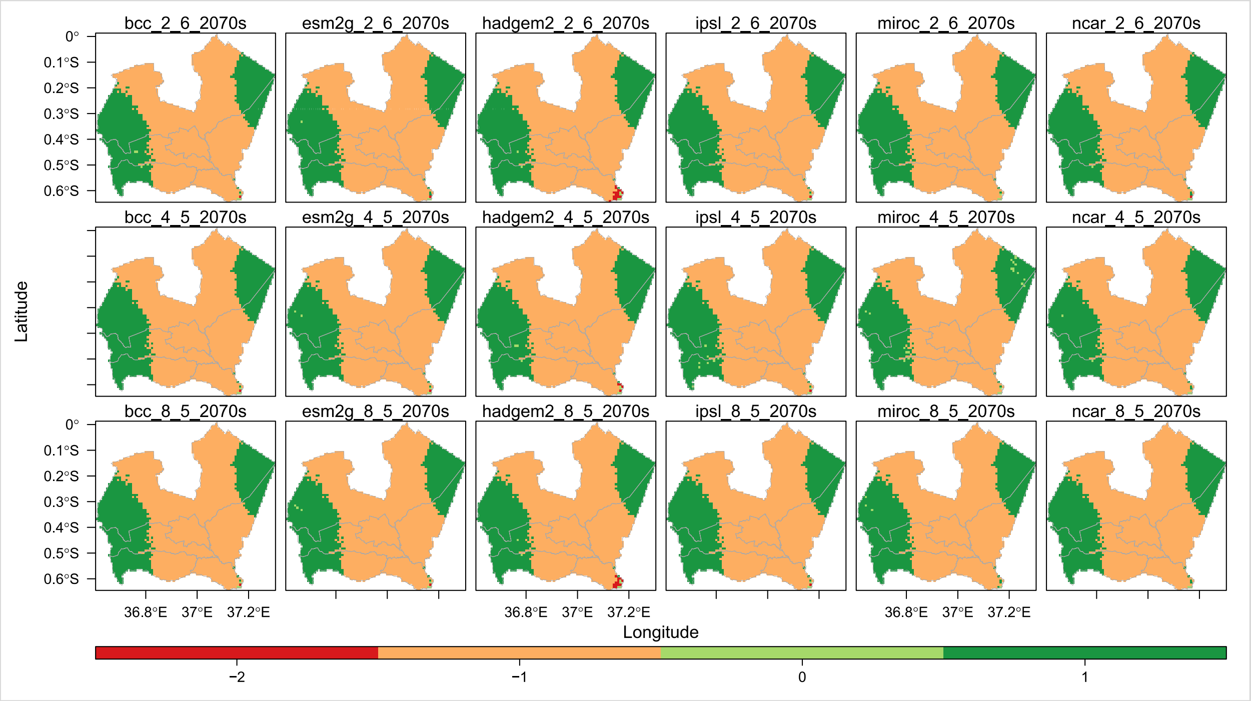

e) *S. campylacanthum* Hochst. ex A. Rich.

Potential range size changes - 2050

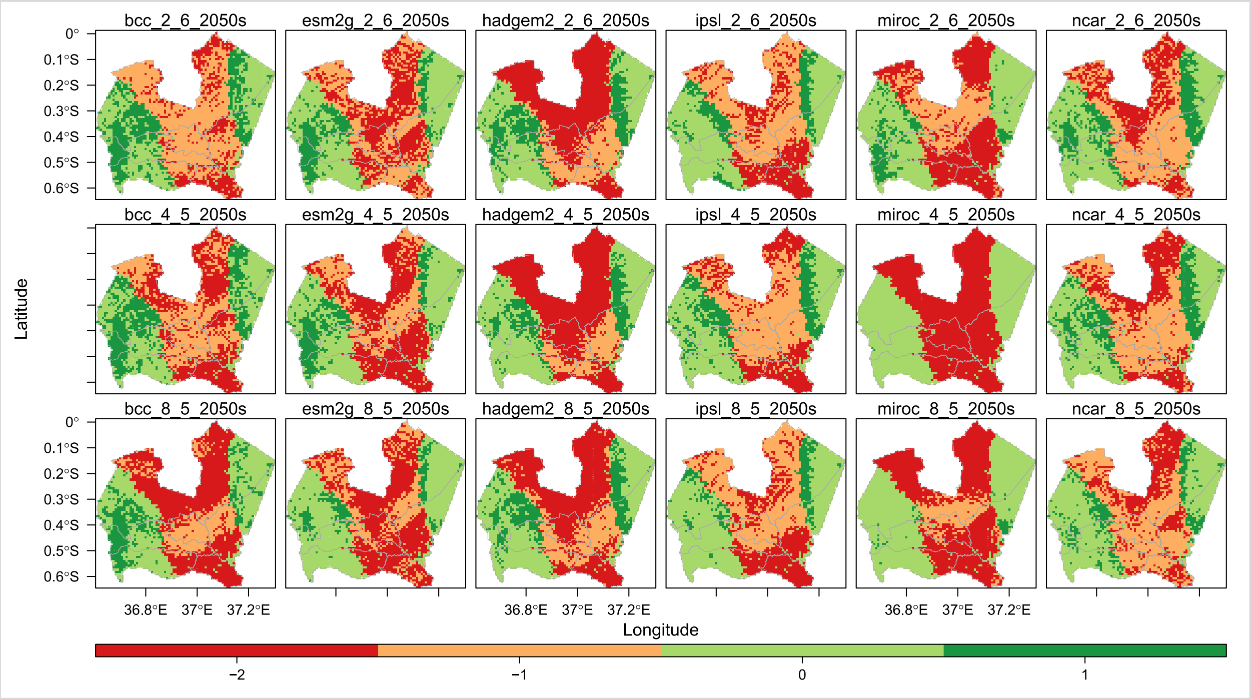

Potential range size changes - 2070

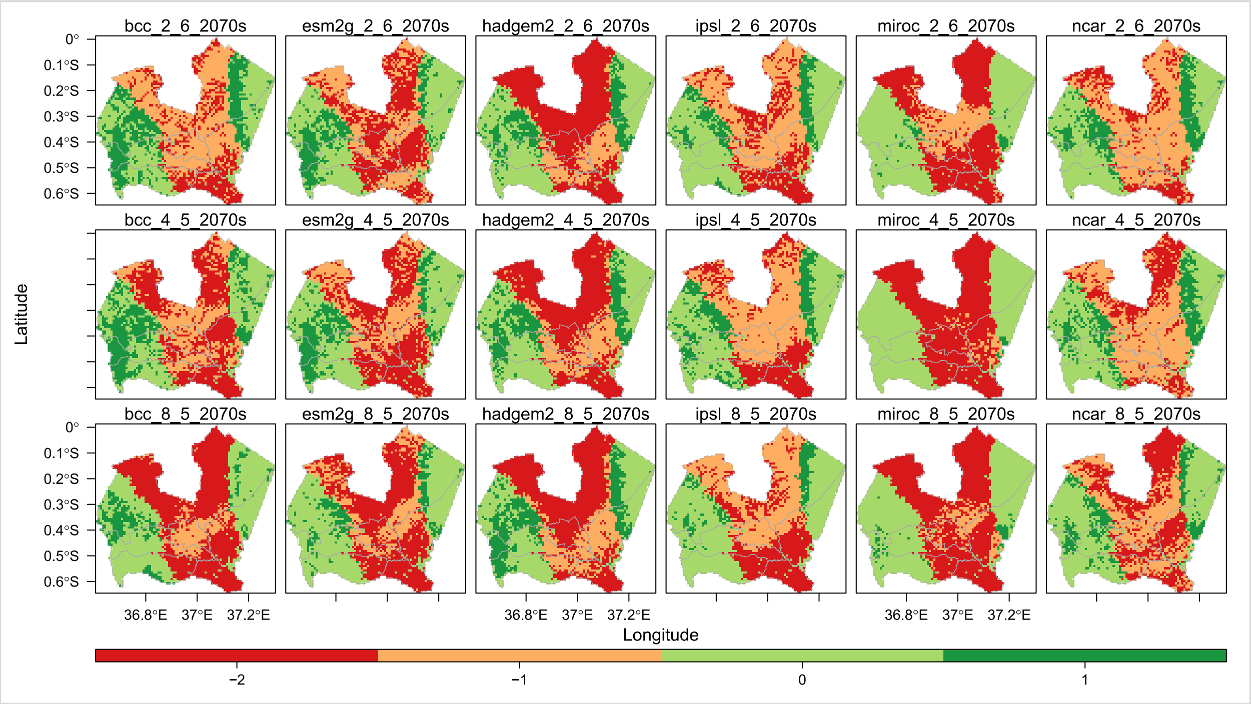

Fig 2. Predicted potential habitat gain, loss and overall habitat changes obtained from a simple average of the outputs from individual GCM data.

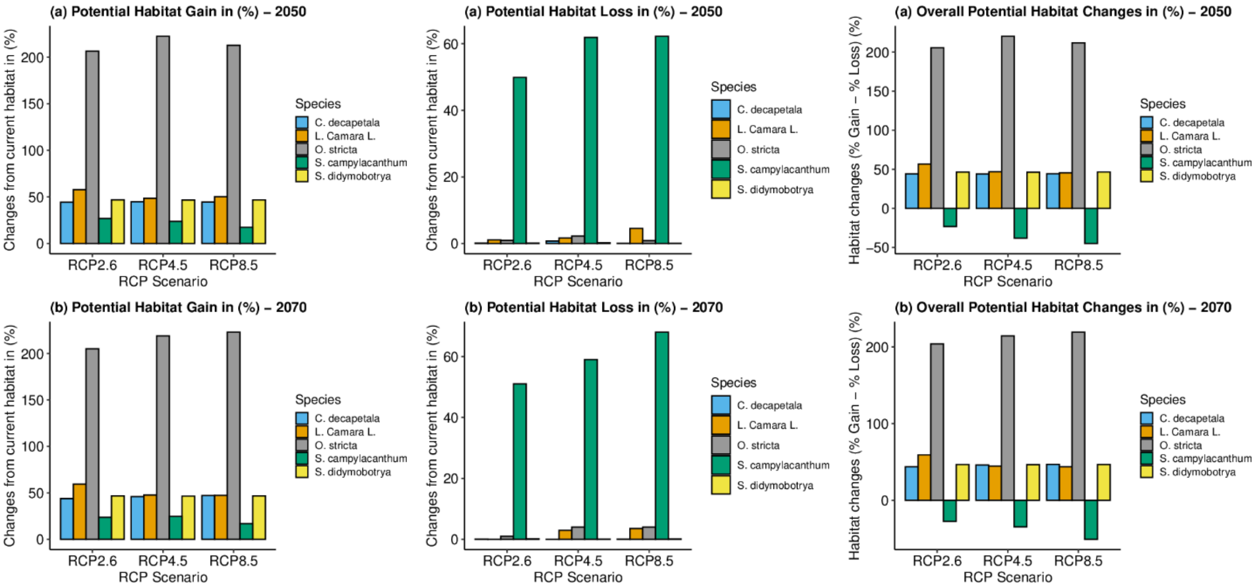

Supplement: S6 Appendix — (PDF) [file pone.0275360.s006.pdf]
